# Defect Tolerance via External Passivation in the Photocatalyst SrTiO_3_:Al

**DOI:** 10.1021/jacs.5c07104

**Published:** 2025-06-23

**Authors:** Kanta Ogawa, Seán R Kavanagh, Fumiyasu Oba, Aron Walsh

**Affiliations:** † Department of Materials, 4615 Imperial College London, London SW7 2AZ, United Kingdom; ‡ Materials and Structures Laboratory, Institute of Integrated Research, 427400 Institute of Science Tokyo, R3-7, 4259 Nagatsuta, Midori-ku, Yokohama 226-8501, Japan; § Laboratory for Materials and Structures, Institute of Innovative Research, Tokyo Institute of Technology, R3-7, 4259 Nagatsuta, Midori-ku, Yokohama 226-8501, Japan; ∥ Harvard University Center for the Environment, Harvard University, Cambridge, Massachusetts 02138, United States

## Abstract

The efficiency of solar-to-energy conversion in semiconductors is limited by charge carrier recombination, often via defect-induced gap states. Although some materials exhibit an intrinsic defect tolerance that avoids fast recombination channels, there are few examples among metal oxides. We investigate the water splitting photocatalyst SrTiO_3_, where photocatalytic performance is enhanced by extrinsic Al doping. We propose that defect tolerance emerges through a passivation effect that effectively eliminates in-gap states and nonradiative recombination. First-principles defect calculations show that oxygen vacancies are the primary defect species in SrTiO_3_ under oxygen-poor synthetic conditions, which provide in-gap states that are active for carrier capture. Al substitutions are preferred at Ti sites adjacent to the oxygen vacancy, forming [*V*_O_-Al_Ti_] defect complexes. As the oxygen vacancy in-gap state is derived from Ti 3d–Ti 3d interactions across the vacancy, substituting Ti with Al deactivates this interaction and eliminates the in-gap state. The absence of valence d orbitals in Al is key for in-gap state reduction, as supported by the consideration of other dopants such as Sc. Our study illustrates how an orbital-wise understanding of defect states can enable doping strategies to achieve defect tolerance in materials like SrTiO_3_, paving the way for improved solar-to-energy conversion.

## Graphical abstract

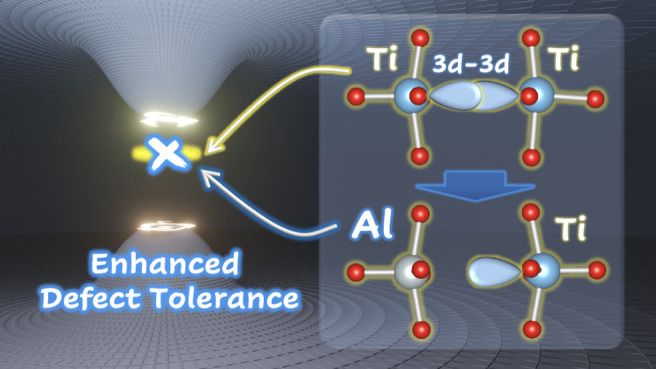

## Introduction

Strontium titanate (SrTiO_3_) has long been a prototype water splitting photocatalyst for clean H_2_ production.^1–3^ Various attempts have been made to enhance catalytic activity, including doping,^4–6^ morphology tuning,^7–9^ and surface modification.^10–13^ The most striking enhancement was achieved with Al doping and appropriate surface modifications, yielding more than 90% quantum efficiency for water-splitting under ultraviolet irradiation.^14,15^ Here, most of the photoexcited carriers are not annihilated by carrier recombination but split water into H_2_ and O_2_ without external electric fields.^15^ This success opened a new chapter toward practical water splitting photocatalysis.^16^

The proposed role of Al doping is to reduce the density of in-gap trap states, believed to be Ti^3+^ states. These trap states provide recombination channels for photogenerated carriers,^6^ as supported by X-ray photoelectron spectroscopy and electronic structure theory.^17^ This is in contrast to the typical role of extrinsic doping in semiconductors, which often generates dopant states and/or charge carriers.^18–22^ The role of Al^3+^ has been linked to its lower valency than the host B-site Ti^4+^, given the photocatalytic activity of SrTiO_3_ can be also improved by other lower oxidation state dopants such as Ga^3+^ and Mg^2+^ but deteriorated by Ta^5+^.^6,23^ However, the mechanistic origin, including how the Ti^3+^ trap state is suppressed by Al doping, and the link to lower valency, remains unresolved.

In this study, we find that the defect tolerance enabled by Al doping of SrTiO_3_ emerges as a passivation effect. Following a first-principles assessment of the intrinsic defect chemistry of SrTiO_3_, we discuss the origins of defect trap states based on their orbital interactions. The dominant deep trap is the Ti^3+^ state derived from the oxygen vacancy. This state is passivated by Al doping through the formation of a [*V*_O_-Al_Ti_] complex, which deactivates the orbital interactions between adjacent Ti-3d states. The absence of valence d orbitals, rather than the lower valency of Al, is found to be key to this behavior.

The distinctive role of Al doping in SrTiO_3_ highlights that defect tolerance can be engineered. Defect tolerance, linked to the avoidance of deep trap states^24^ or nonradiative recombination channels,^25,26^ is usually linked to the fundamental properties of the host crystal, such as an antibonding-type upper valence band^27–32^ or bonding-type lower conduction band.^31–34^ SrTiO_3_ is not “intrinsically” defect tolerant from this viewpoint. Rather, wide-gap semiconductors like SrTiO_3_, with a band gap larger than 1.6 eV, have been less successful due to inherent high (low) ionization potential (electron affinity) and/or high ionicity.^35^ The present study demonstrates the potential for defect-state engineering via doping to passivate intrinsic in-gap defect states and suppress carrier recombination.

## Results and Discussion

### Electronic Structure of SrTiO_3_

Cubic SrTiO_3_ has a typical ABX_3_ perovskite crystal structure with Sr on the cuboctahedral A-site and Ti on the octahedral B-site (Figure 1a). The electronic structure of pristine SrTiO_3_ was calculated using the Heyd–Scuseria–Ernzerhof (HSE06) hybrid exchange-correlation functional^36^ (Table S1). The calculated direct (3.72 eV) and indirect (3.34 eV) band gaps are in reasonable agreement with experimental values (3.75 and 3.25 eV, respectively).^37^ Bonding and antibonding interaction between Ti and O give rise to the valence and conduction bands respectively, while the orbital interactions at the valence band maxima (VBM) and conduction band minima (CBM) are primarily nonbonding (Figure S1).^38^ This electronic structure does not imply intrinsic defect tolerance, although the nonbonding band edges may be less harmful than the bonding VBM and antibonding CBM for avoiding deep traps.

**Figure 1:**
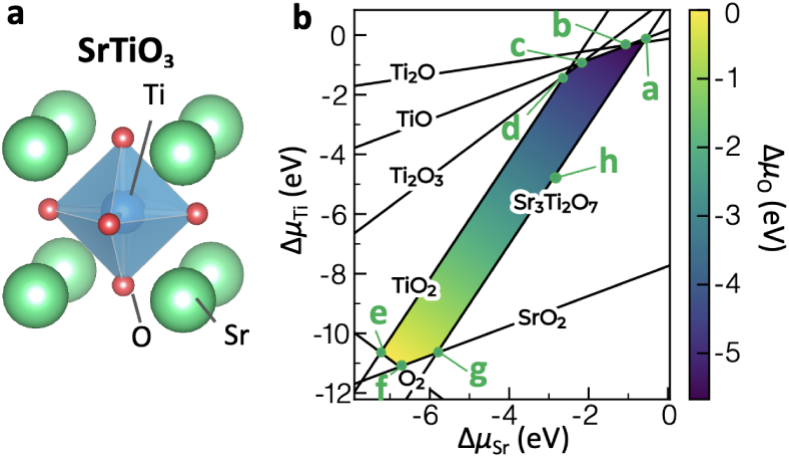
(a) Crystal structure of the cubic perovskite phase of SrTiO_3_. (b) Sr–Ti–O ternary chemical potential diagram showing the stable region of SrTiO_3_ due to the limits imposed by competing phases.

The phase stability of SrTiO_3_ was determined by calculating the total energy of the competing phases within the Sr–Ti–O chemical space using doped package^39^ (Table S2). All possible competing phases (stable and metastable ones within a tolerance of 0.1 eV/atom) were obtained from the Materials Project^40^ database and their energies were computed using the HSE06 hybrid functional, in order to determine the stable region of the elemental chemical potentials (Δμ_Sr_, Δμ_Ti_, Δμ_O_), where each Δμ_i_ represents the chemical potential relative to the standard chemical potential (i.e., μ_Sr_°, μ_Ti_°, μ_O_° = 1/2 μ_O_2__°, respectively). The sum of Δμ_Sr_, Δμ_Ti_, and Δμ_O_ is given by the formation energy of SrTiO_3_, Δ*E*_*f*_ (SrTiO_3_), namely,

$$\Delta \mu_{Sr}+\Delta \mu_{Ti}+3\Delta \mu_{O}=\Delta E_{f}\left({\mathrm{SrTiO}}_{3}\right)$$

In addition, Δμ_Sr_, Δμ_Ti_, Δμ_O_ should not stabilize other competing phases such as TiO_2_, namely,

$$\Delta \mu_{Ti}+2\Delta \mu_{O}\leq \Delta E_{f}\left(TiO_{2}\right)$$

Such constraints determine the chemical potential ranges for Δμ_Sr_, Δμ_Ti_, Δμ_O_ that stabilize SrTiO_3_, as represented by the colored area in Figure 1b defined by the seven limit conditions (**a**–**g**). Here, we see that SrTiO_3_ occupies quite a wide range of chemical potentials in the Sr–Ti–O chemical space, with Δμ_Sr_ and Δμ_O_ varying by ∼6 eV and Δμ_Ti_ varying by ∼10 eV within the stable region of SrTiO_3_.

### Defect Formation Energy and Concentration

Within the supercell formalism for computing defect energetics, the formation energy of a defect *X* in charge state *q*, $E_{X,q}^{f}\left(\varepsilon_{F},\mu \right)$ , is given as^41–43^

1 $$E_{X,q}^{f}\left(\varepsilon_{F},\mu \right)=E_{X,q}-\left[E_{H}+\underset{i}{\sum }n_{i}\mu_{i}+q\cdot \left(-\varepsilon_{F}\right)\right]+E_{corr}\left(q\right)$$

Here, *E*_*X*,*q*_ is the total energy of the supercell containing the defect, while *E*_*H*_ is the energy of the corresponding pristine bulk supercell. $\underset{i}{\sum }n_{i}\mu_{i}$ represents the energy of *n_i_* atom of kind *i* from a reservoir with chemical potential *μ_i_*. Analogously, −*qε*_F_ accounts for the energy of charge *q* from the reservoir having the given chemical potential of electrons, i.e., the Fermi level *ε*_F_. The last term *E*_corr_(*q*) is the charge-state dependent correction to account for spurious electrostatic interactions arising from the finite supercell size. The extended Freysoldt–Neugebauer–Van de Walle (eFNV) correction scheme was employed in the present study.^44,45^

While *μ*_*i*_ and *ε*_F_ are variable, *E_X,q_*, *E_H_* should be calculated carefully for an accurate determination of the defect formation energy. Herein, the HSE06 hybrid functional was employed. In addition, *E_X,q_* depends on the calculated (relaxed) defect structure, which is sensitive to the choice of initial geometry.^46^ The standard approach, based on local optimization of a defect-containing supercell, sometimes misses the true ground state by falling into a local minimum.^47^ To avoid this, we employed a robust yet efficient searching method, where the initial structures are generated through chemically guided bond distortions and rattling (the ShakeNBreak approach).^48,49^ Effective sampling of the potential energy surface enables us to identify energy-lowering structural reconstructions.

By calculating the formation energy of all the possible native defects including vacancies, antisites, and interstitials (Table S3), a defect formation energy diagram at a certain chemical potential condition is obtained (e.g., Figure 2b for the oxygen poor limit). From this diagram, we can estimate the concentration of defect *X* in charge state *q* (*C*_*X*,*q*_) at a certain temperature *T*. Since *C_X,q_,* as well as the concentrations of free holes and electrons (*p* and *n*), depend on *ε*_F_ , the charge neutrality condition self-consistently determine *ε*_F_ , and thus *C_X,q_,* , *p*, and *n* (see the Experimental Section for details).^50,51^

**Figure 2:**
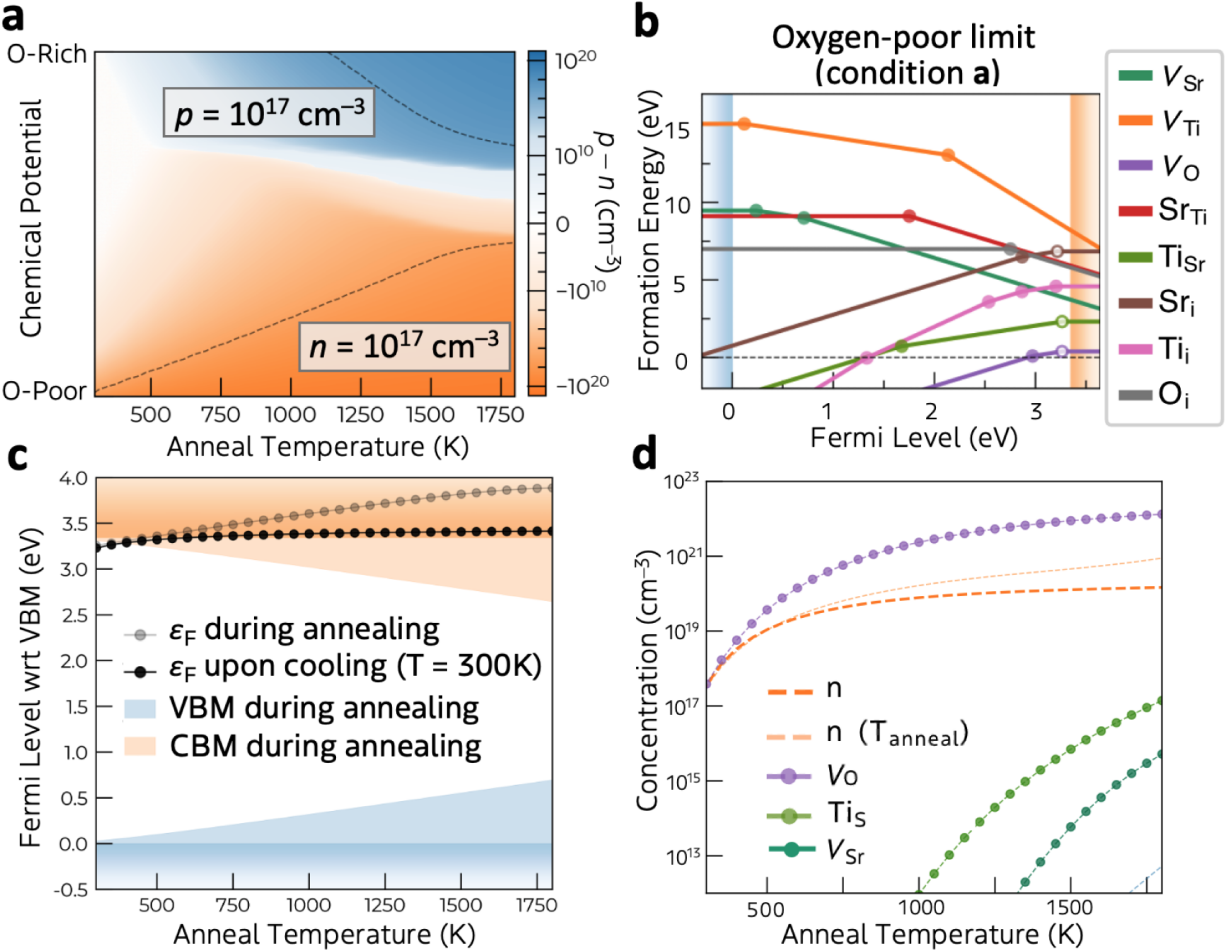
(a) Calculated carrier concentration in undoped SrTiO_3_ at room temperature, as a function of annealing temperature and chemical potential conditions. Contour lines correspond to the carrier concentration of 10^17^ cm^–3^. (b) Formation energies and transition levels (filled circles) of native defects in SrTiO_3_ at the oxygen-poor limit (condition a: Δμ_O_ = −5.67, Δμ_Sr_ = −0.62, Δμ_Ti_ = −0.25 eV), where shallow donor levels associated with electronic states inheriting host band edge orbital characteristics are designated by open circles. (c) Calculated self-consistent Fermi level positions in undoped SrTiO_3_ during annealing (gray) and upon cooling to room temperature (300 K; black), assuming the oxygen-poor limit. (d) Room temperature carrier and defect concentrations as a function of annealing temperature, assuming the oxygen-poor limit.

Figure 2a shows the carrier concentration in undoped SrTiO_3_ as a function of anneal temperature and chemical potential during the synthesis process. The frozen defect approximation is applied to mimic a synthetic condition where the material is annealed under elevated temperatures and cooled rapidly to room temperature.^51,52^ Defects are formed at the elevated annealing temperature but remain during cooling due to the assumed large kinetic barriers for diffusion and annihilation. The temperature dependence of the band gap^53^ (at annealing temperatures) was also taken into account. Clearly, n-type conditions (orange) dominate over p-type ones (blue). Higher electron densities are achievable compared to hole densities, showing the native n-type preference of undoped SrTiO_3_. Although p-type conductivity is observed for undoped-SrTiO_3_ at high temperature and under high oxygen partial pressure,^54^ consistent with the calculated carrier polarity, the p-type regime is limited because of the quenched-in oxygen vacancy population even at a low temperature.^55^ Note that the highly active SrTiO_3_:Al photocatalyst is synthesized using a flux-assisted method, where the crystal grows in the molten salt of SrCl_2_ at 1423 K.^14,15^ In this growth condition, the oxygen chemical potential should be poor due to the lack of contact with O_2_ molecules in air and a high temperature. Indeed, SrTiO_3_:Al exhibits n-type character with an estimated electron concentration (*n*) of 1.7 × 10^17^ cm^–3^ from its Mott–Schottky plot.^56^ Therefore, we mainly focus on oxygen-poor conditions hereafter.

The defect formation energy diagram at the oxygen-poor limit (condition **a** in Figure 1b) is shown in Figure 2b. The formation energies at other chemical potential limits are described in Figures S2 and S3. Under the oxygen-poor limit condition (condition **a**: Δμ_O_ = −5.67, Δμ_Sr_ = −0.62, Δμ_Ti_ = −0.25 eV) considered in Figure 2c, SrTiO_3_ presents an n-type character with *ε*_F_ near the CBM. Figure 2d shows the defect concentration plotted versus the annealing temperature at the oxygen poor limit. Oxygen vacancy (*V*_O_) is the highest concentration defect species, followed by titanium-on-strontium antisite (Ti_Sr_). The latter is suppressed under Sr-rich condition (Figure S4).

As established from experiment,^17,57^ the oxygen vacancy concentrations in SrTiO_3_ under O-poor conditions and at high annealing temperatures are extremely high (>10^21^ cm^–3^; Figure 2d), approaching percent-level concentrations relative to the oxygen sites (having a bulk site concentration of ∼ 5 × 10^22^ cm^–3^). At these high concentrations, there is likely to be non-negligible *V*_O_–*V*_O_ interactions,^58^ implying that the predicted defect concentrations should be treated semiquantitatively. Still, such effects would not alter the conclusion that the oxygen vacancies are the dominant defect species under O-poor conditions.

### Nature of the *V*_O_ Deep State

As expected, oxygen vacancies (*V*_O_) have the lowest formation energy in the oxygen-poor region, with the dominant charge state depending on *ε*_F_ (Figures 2b and S5). The *ε*_F_ position where the defect formation energies for different charge states (*q*_1_ and *q*_2_) are equal is called a charge transition level *ε*(*q*_1_/*q*_2_). These are also known as carrier trap levels because they define the thermal ionization energies or electron affinities of the donor/acceptor. The charge transition of *V*_O_ from +2 to +1, i.e., *ε*(+2/+1), occurs at 0.37 eV, and from +1 to 0 at 0.08 eV below the CBM (Table S5). The former means that 0.37 eV is required to thermally excite one electron from *V*_O_^+1^ to form *V*_O_^+2^, defining the trap energy of an electron at *V*_O_^+2^ (Figure S5). The present result suggests that the oxygen vacancy is a double donor where one electron is easily released to the conduction band while the second tends to be trapped within the band gap, consistent with the previous experiment^59,60^ and calculation results.^61–64^ The calculated transition levels are in good agreement with those estimated by analyzing the temperature-dependent transport behavior, where ionization energies of 0.3 eV for *ε*(+2/+1) and <3 meV for *ε*(+1/0) were reported.^65^

These charge transition levels reflect the underlying difference in the single-particle electronic bands. As shown in Figure 3, while the closed-shell *V*_O_^+2^ does not induce an in-gap state, *V*_O_^+1^ produces a localized state below the CBM, which causes the in-gap transition level in Figures 2b and S5. In this study, we show that Al doping to SrTiO_3_ reduces trap states. Before investigating the effect of Al doping, we examine the origin of trap states at oxygen vacancies by analyzing the single-particle electronic DOS for each *V*_O_ charge state.

**Figure 3:**
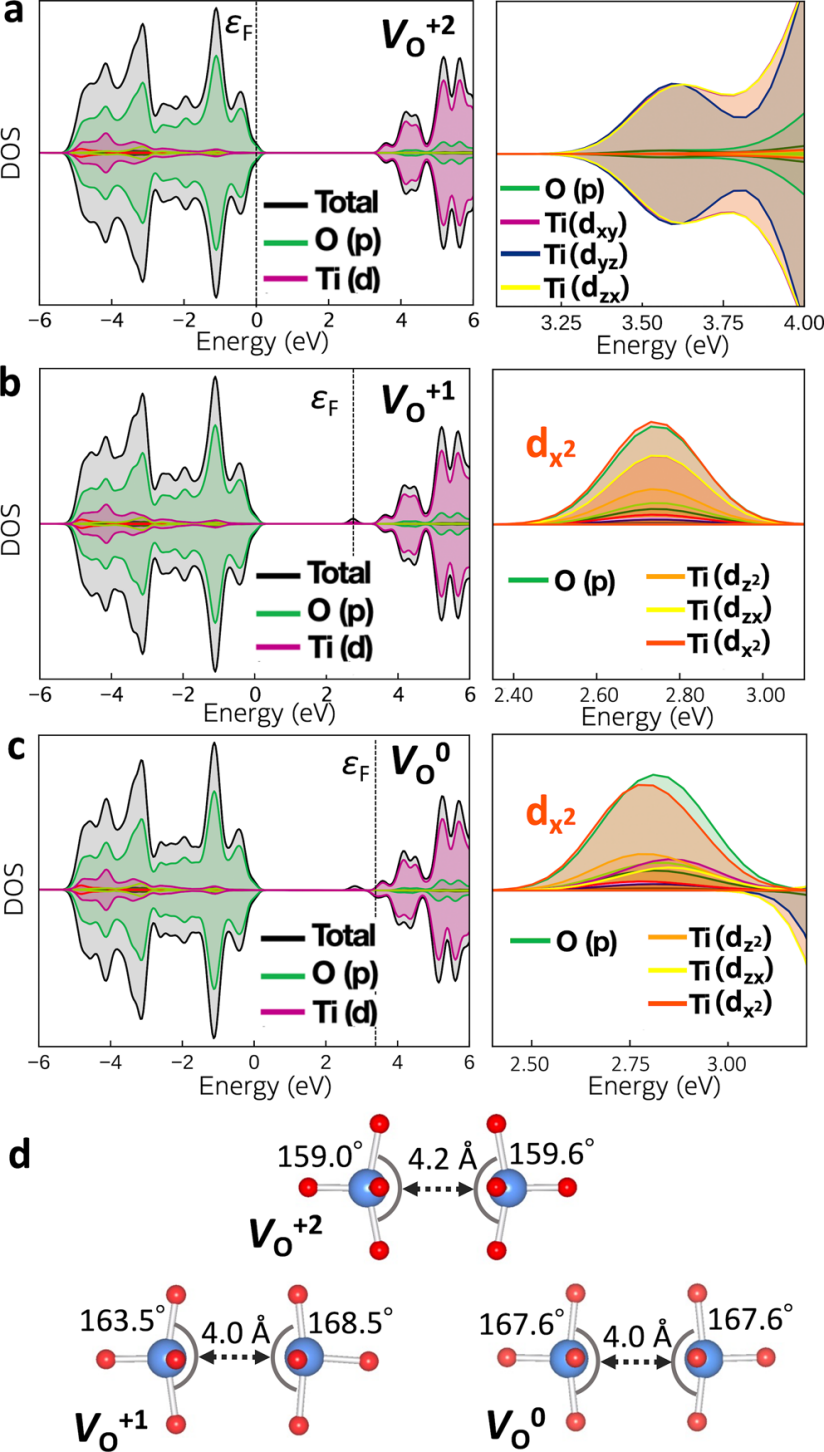
Electronic densities of states for SrTiO_3_ supercells containing (a) *V*_O_^+2^, (b) *V*_O_^+1^, and (c) *V*_O_^0^ with coordination environment of Ti atoms adjacent to the oxygen vacancy (d). The upper and lower panels in each DOS represent the majority and minority spins, respectively. The original Ti–Ti distance in the absence of oxygen vacancy is 3.9 Å.

Figure 3a shows the DOS of the supercell with the fully ionized *V*_O_^+2^, which represents the removal of O^2–^ and thus there are no excess electrons in the system. The lower conduction band is derived from Ti-3d t_2g_ orbitals (d_*xy*_, d_*yz*_, d_*zx*_). The ground state *V*_O_^+2^ structure involves two Ti atoms moving away from the oxygen vacancy, resulting in a Ti–Ti distance of 4.2 Å (Figure 3d). A similar geometry is found in other computational studies.^62,66,67^ The removal of an oxide ion triggers a corresponding atomic displacement with two neighboring Ti ions repelled, due to the cleaved electrostatic attraction and orbital interaction. The deformation stabilizes the low coordination Ti sites via Ti 3d–O 2p interaction to form bonding–antibonding combinations directed away from the vacancy (as in the second-order Jahn–Teller effect) as shown in Figure 4a.^66,68^

**Figure 4:**
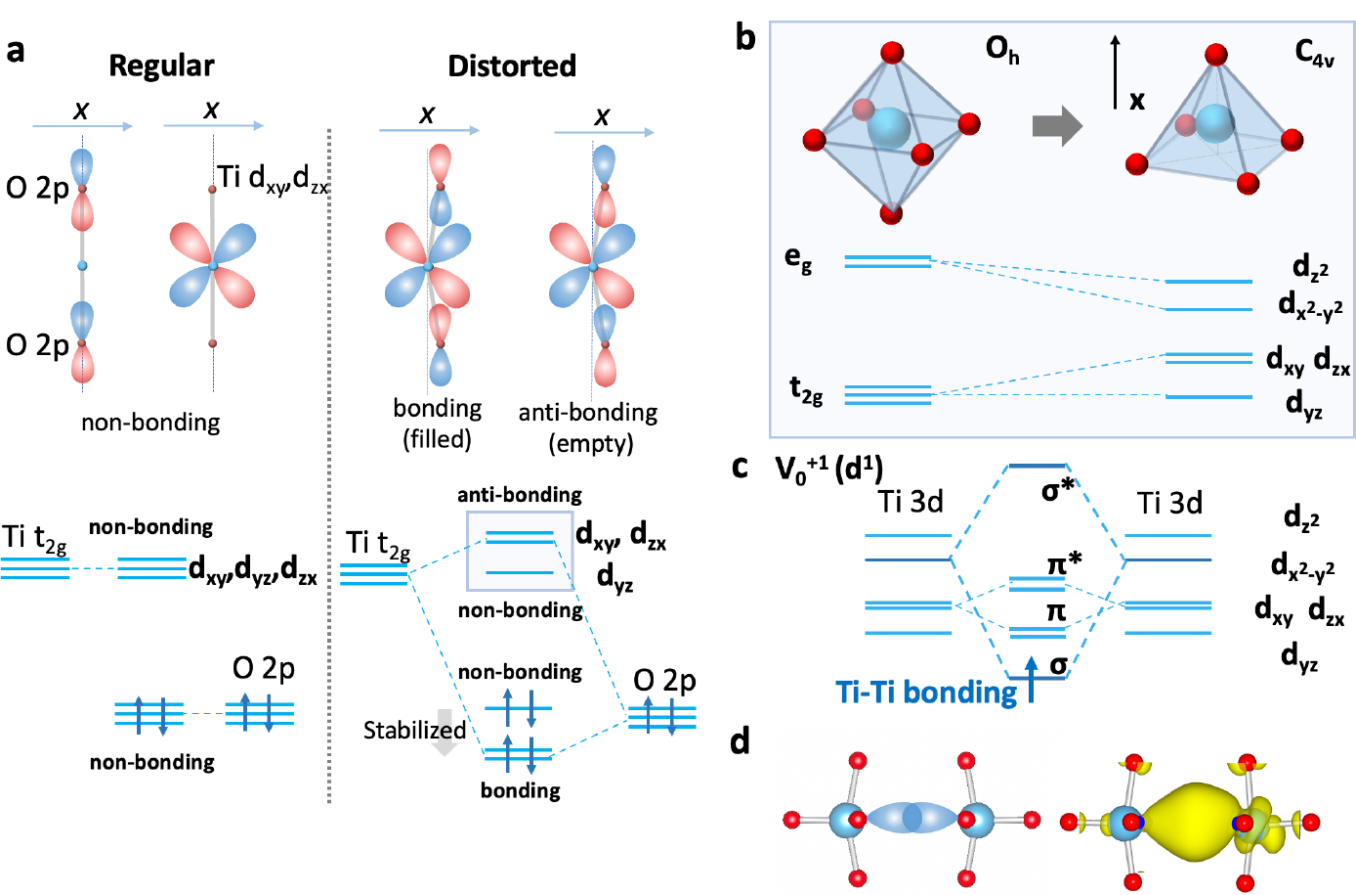
(a) Illustration of the band-edge orbital interaction in SrTiO_3_. In a regular octahedral environment, the band edge O 2p and Ti 3d states are nonbonding. The oxygen vacancy allows for a distorted environment, which is stabilized by Ti–O bonding–antibonding interaction. (b) Resolved degeneracy of d-orbitals through the coordination environment change from *O_h_* to *C*_4*v*_ accompanied by the oxygen vacancy. (c) Orbital interaction between two Ti atoms across the oxygen vacancy. The d*_x^2^_* orbitals directed toward each other interact to form σ and σ* states, with the former corresponding to the in-gap trap state. (d) Schematic illustration of Ti 3d–Ti 3d interaction across the oxygen vacancy and calculated electron density map of the *V*_O_^+1^-induced in-gap state shown in Figure 3b (2.3–3.2 eV) is also shown (at an isosurface value of 2 × 10^–3^ e/Å^3^). The uneven charge density distribution between the two Ti cations may arise from the competition between the orbital interaction (c), which favors charge distribution, and the Coulombic interaction, which favors electron localization.

In contrast to the fully ionized defect, *V*_O_^+1^ and *V*_O_^0^ exhibit occupied in-gap states associated with Ti^3+^ (Figure 3b,c). This is consistent with the experimental observations that the oxygen vacancy reduces Ti^4+^ to Ti^3+^ in SrTiO_3_.^17^ This in-gap state is contributed by Ti 3d_*x* ^2^–*y* ^2^_ from e_g_, which cannot be explained by considering the coordination environment of only one Ti atom. As shown in Figure 4b, at the distorted Ti environment with an oxygen vacancy (with *C*_4*v*_ point symmetry), Ti 3d*_yz_* from the t_2g_ set is the lowest energy molecular orbital. There are two Ti atoms adjacent to one oxygen vacancy, however. Here, the e_g_ orbitals (d_*x* ^2^–*y* ^2^_) of the two Ti atoms are directed toward each other along the *x*-axis, to form the σ bonding–antibonding interaction (Figure 4c) as suggested previously.^69–71^ For *V*_O_^+1^ and *V*_O_^0^, the additional electrons occupy the bonding state, stabilizing the Ti–Ti interaction. When the interaction is strong enough, the σ bonding state (d_*x* ^2^–*y* ^2^_) is lower than the 3d*_yz_* (i.e., the original CBM), yielding the in-gap state. On the other hand, the Ti–Ti interaction is less favorable for *V*_O_^+2^ because of the absence of electrons that can occupy the σ state as evidenced by greater Ti–Ti separation, making *V*_O_^+2^ free from the in-gap state. While the cleavage of the O–Ti bonds at the vacancy site and resulting outward Ti displacements narrows the O–Ti–O angle, the Ti–Ti interaction favors reduced Ti–Ti distances and acts to widen the O–Ti–O angle (Figure 3d), giving competing effects. Although some computational studies report an O–Ti–O angle exceeding 180°,^63^ such a structure is not stabilized with the present computational setup. Another possible state is one electron mostly localized on one Ti atom.^63^ However, this polaronic state is slightly higher in energy than the Ti–Ti bonding state in the present calculation (Figure S6). From these results, we conclude that the origin of the in-gap state is the Ti 3d–Ti 3d bonding interaction across the oxygen vacancy. An analogue can be seen in the lead halide perovskites, where Pb 6p–Pb 6p σ bonding across the halide vacancy causes an in-gap state.^72^

This in-gap state formed with the oxygen vacancy is believed to deteriorate the activity of SrTiO_3_ photocatalysts by trapping photoexcited electrons.^6,15,17,23^ We evaluated the nonradiative carrier capture process when SrTiO_3_ is subject to above band gap illumination within the framework of multiphonon emission,^73^ i.e.,

2 $$V_{O}^{+2}\overset{h\nu }{⇌}V_{O}^{+2}+e^{-}+h^{+}\overset{-\hbar \omega }{⇌}V_{O}^{+}+h^{+}\overset{-\hbar \omega }{⇌}V_{O}^{+2}$$

where the excess electronic energy provided by light absorption (*hν*) is thermally emitted through phonons (ℏω) . The potential energy surface (PES) between *V*_O_^+2^ and *V*_O_^+^ was calculated along the one-dimensional configuration coordinate *Q* defined as

3 $$Q^{2}=\underset{\alpha }{\sum }M_{\alpha }\Delta R_{\alpha }^{2}$$

where $M_{\alpha }$ and $\Delta R_{\alpha }$ are the mass and the displacement of an atom α between the two configurations (Figure 5a). Based on these PES, the nuclear wave function overlaps were obtained via the 1D Schrödinger equation.^74,75^ Combined with the electron–phonon coupling coefficient calculated within the static coupling approach,^73,76^ we obtained the carrier capture cross sections $\sigma_{e/h}$ . They show weak temperature dependence in the range up to 500 K (Figure S7). The small activation energy barrier (Δ*E*_*e*_) and the Coulombic attraction between the positively charged *V*_O_^+2^ and free electrons result in a large electron capture coefficient (*C*_*e*_) of 5.6 × 10^–8^ cm^3^ s^–1^ and cross-section (σ_*e*_) of 3.6 × 10^–15^ cm^2^ at room temperature (Table S6), suggesting fast electron capture by the oxygen vacancy. On the other hand, the hole capture is much slower with a capture coefficient (*C*_*h*_) of 9.5 × 10^–23^ cm^3^ s^–1^ and cross-section (σ_*h*_) of 7.4 × 10^–30^ cm^2^. These results are consistent with an experimental observation of a long hole lifetime even after the fast electron trapping producing Ti^3+^, as revealed by transient absorption measurements.^77^ The trapping not only immobilizes (i.e., localizes) the electrons but also lowers their energetic potential. Given the relatively small overpotential for the water reduction, i.e., the CBM position vs RHE of SrTiO_3_ (0.4–0.8 eV),^78,79^ the energy loss by trapping (∼0.4 eV) can be extremely detrimental for water splitting photocatalytic activity by limiting the H_2_O reduction process.

**Figure 5:**
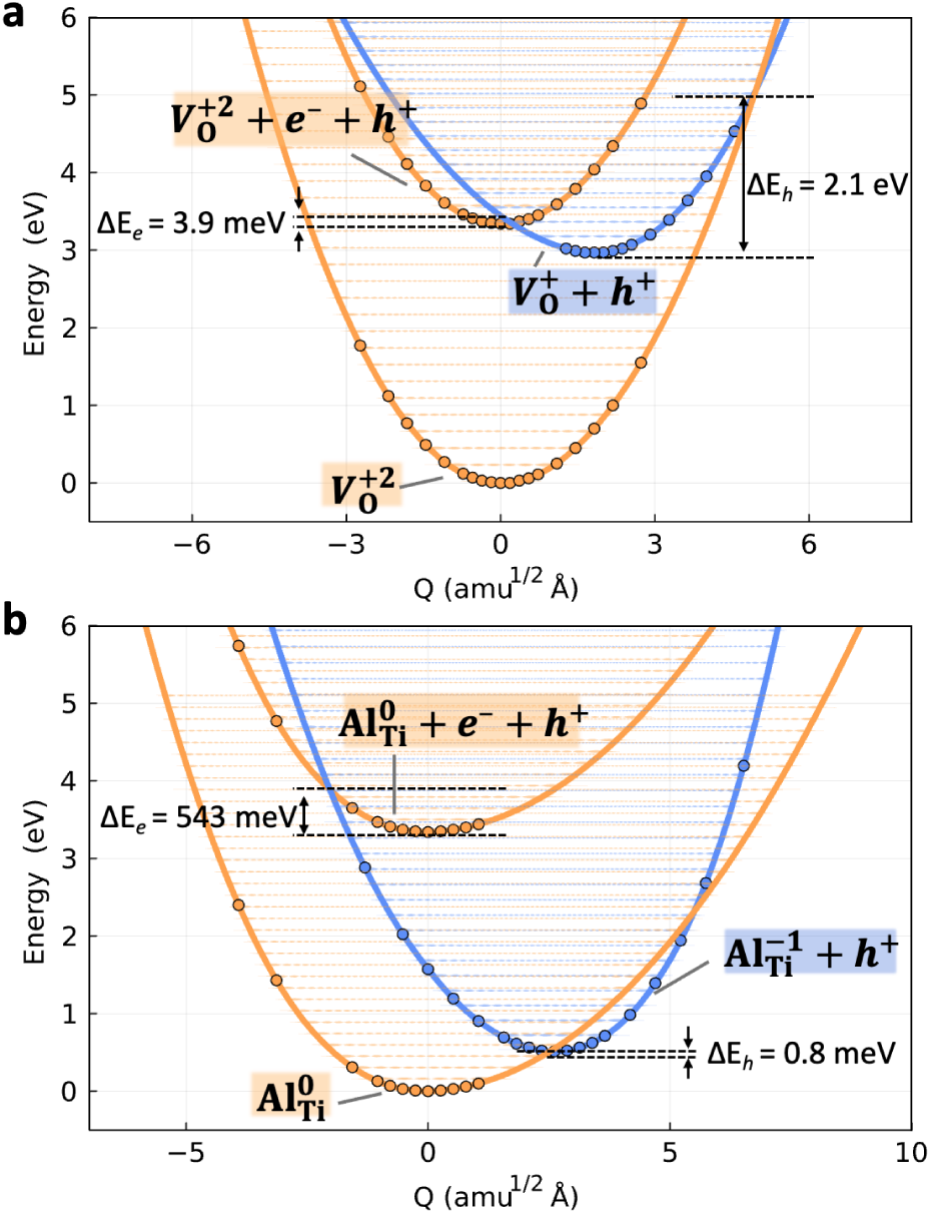
Potential energy surface (PES) for charge transition between (a) *V*_O_^+2^ and (b) *V*_O_^+^ and Al_Ti_^0^ and Al_Ti_^–1^ in SrTiO_3_. The transition from the upper orange PES (*V*_O_^+2^ (Al_Ti_^0^) + e^–^ + h^+^) to the middle blue PES (*V*_O_^+^ (Al_Ti_^–1^) + h^+^) corresponds to electron capture by *V*_O_^+2^ (Al_Ti_^0^), while that from the middle blue to the lower orange PES (*V*_O_^+2^ (Al_Ti_^0^)) corresponds to hole capture by *V*_O_^+^ (Al_Ti_^–1^). Δ*E*_h/e_ denotes the activation energy barrier to hole/electron capture.

### Effect of Al Doping

There are several mechanisms through which Al can reduce the trap density in SrTiO_3_. One indirect route is to lower the Fermi level through Al^3+^ replacement of Ti^4+^, which can affect the self-consistent concentration of *V*_O_. Another mechanism involves the direct modification of the electronic structure and defect chemistry at the *V*_O_ site.

Al can be substitutional on Sr or Ti sites (Al_Sr_, Al_Ti_), or may form an interstitial (Al_i_). The formation energies of Al_Sr_, Al_Ti_, and Al_i_ are calculated for the oxygen-poor limit (Figure 6a). Considering the flux synthesis of SrTiO_3_:Al at ∼1400 K in the molten salt of SrCl_2_ (Figures S8 and S9), the Sr-rich condition with Δμ_O_ = −3 eV is also investigated (denoted as condition **h**; Figure 1b). Δμ_Al_ is determined by SrAl_2_O_4_ as a competing phase, assuming an Al-rich condition for both cases. When *ε*_F_ is near the CBM, the main Al-related species is Al_Ti_^–1^, consistent with the experimentally intended species.

**Figure 6:**
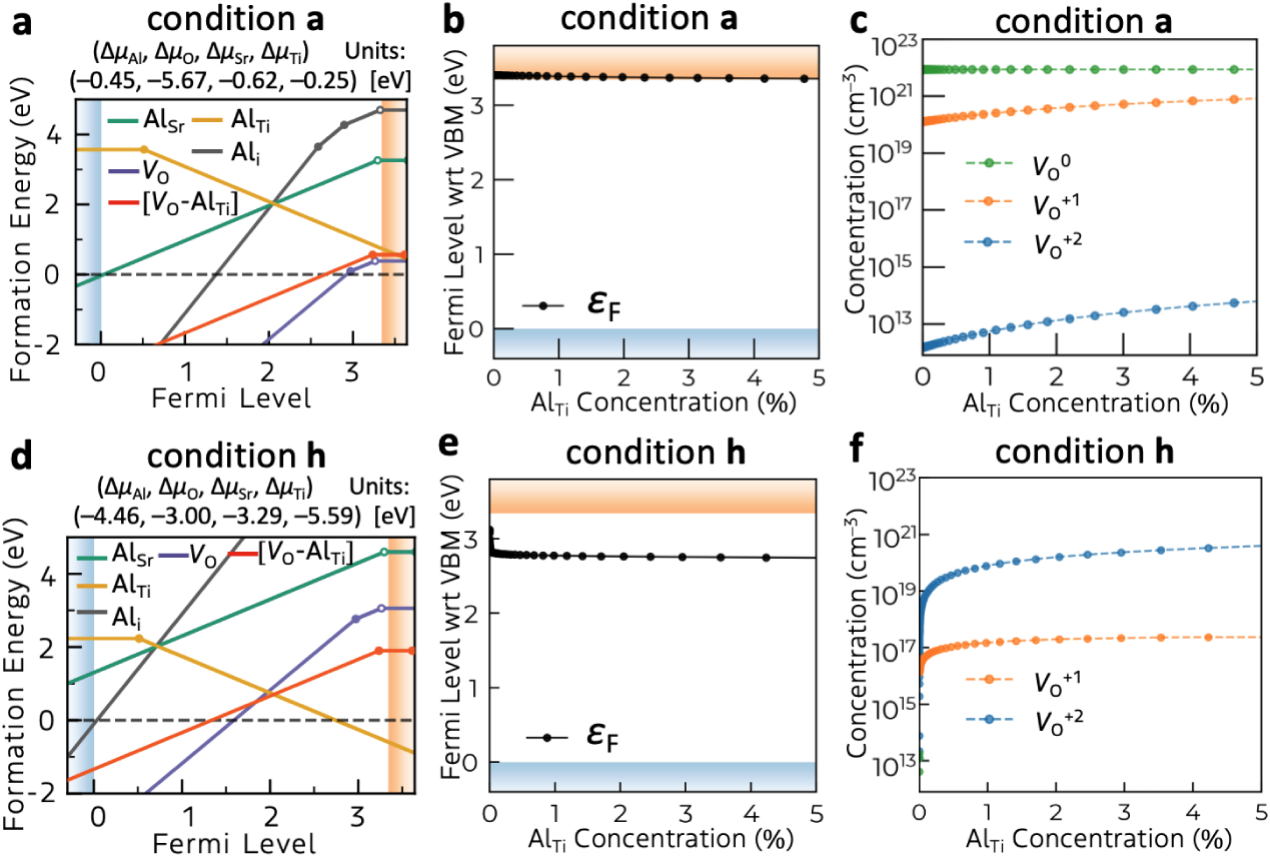
(a, d) Formation energies and transition level diagrams of Al dopant species, oxygen vacancy, and the defect complex (oxygen vacancy + Al substitution on Ti) in SrTiO_3_ under condition **a** (oxygen poor limit) and condition **h** (Δμ_O_ = −3 eV). In both cases, Al-rich conditions are assumed, where the upper limits of Δμ_Al_ are set to −0.45 eV and −4.46 eV for conditions **a** and **h**, respectively, given SrAl_2_O_4_ as the bordering phase. (b, e) The effect of Al dopant concentration on the self-consistent Fermi levels. (c, f) The corresponding concentration of each charge state of *V*_O_ when the system is annealed at 1400 K and cooled to room temperature. For reference, the experimental Al dopant concentration in SrTiO_3_:Al is ∼1 at%.

The effect of the Al dopant concentration on *ε*_F_ and the *V*_O_ species is calculated by changing the Δμ_Al_ and, thereby, the corresponding Al concentration at each condition. As shown in Figure 6, under both conditions, Al doping slightly lowers *ε*_F_ because of the electron compensation by the substitutional Al defect (Al_Ti_^–1^). The Fermi level lowering increases the *V*_O_^+1^ and *V*_O_^+2^ concentrations (Figure 6c,f). The experimental dopant concentration is reported to be around 1% atomic ratio to the Ti site.^15,23^ As expected from the negative charge of the main defect species (Al_Ti_^–1^), Al doping increases the total concentration of positively charged *V*_O_ states, which cannot explain the observed in-gap state passivation by Al doping. In addition, Al_Ti_ itself can act as a hole trap (Figure 5b), consistent with photocatalytic activity lowering with excessive Al doping.^14^ Thus, isolated Al_Ti_ cannot explain the increased photocatalytic activity of SrTiO_3_ induced by Al doping.

### Formation of a Defect Complex

Another possibility is that the electronic structure is changed by Al association with *V*_O_.^17,80^ A complex would be stabilized by the Coulombic attraction between positively charged *V*_O_^+1/+2^ and Al_Ti_^–1^, as well as the high concentrations of oxygen vacancies and Al dopants (thus lower entropic cost to complex formation).^52,81^

Here, Al_Ti_ exhibits the lowest formation energy for Al-related point defects when *ε*_F_ is near the CBM (Figure 6). When forming a complex with oxygen vacancies [*V*_O_-Al_Ti_], Al prefers to replace Ti adjacent to the oxygen vacancy (Figure S10). The complex binding energy from two isolated defects, A + B ⇌ AB, is given by

4 $$\Delta E=E^{f}\left(AB\right)-\left(E^{f}\left(A\right)+E^{f}\left(B\right)\right)$$

where *E* ^*f*^(A), *E* ^*f*^(B) and *E* ^*f*^(AB) are the formation energies of isolated defects A, B and a complex AB as determined from eq 1. We consider the following three association reaction retaining the total charge for simplicity:

5 $$V_{O}^{0}+{\mathrm{Al}}_{\mathrm{Ti}}^{-1}⇌{[V_{O}-{\mathrm{Al}}_{\mathrm{Ti}}]}^{-1},\Delta E=-0.29\;\mathrm{eV}$$

6 $$V_{O}^{+1}+{\mathrm{Al}}_{\mathrm{Ti}}^{-1}⇌{[V_{O}-{\mathrm{Al}}_{\mathrm{Ti}}]}^{0},\Delta E=-0.64\;\mathrm{eV}$$

7 $$V_{O}^{+2}+{\mathrm{Al}}_{\mathrm{Ti}}^{-1}⇌{[V_{O}-{\mathrm{Al}}_{\mathrm{Ti}}]}^{+1},\Delta E=-0.90\;\mathrm{eV}$$

The more negative Δ*E* for the higher charge of *V*_O_ is due to the stronger Coulomb interaction. Complex formation is exothermic in all three cases.

The equilibrium constant for complexation can be approximated from the mass-action law:^81^

8 $$K=\frac{C_{AB}}{C_{A}C_{B}}=\frac{N_{AB}}{N_{A}N_{B}}\exp\left(-\frac{\Delta E}{k_{B}T}\right)$$

where *C*_A_, *C*_B_ and *C*_AB_ are concentrations of defects and *N*_A_, *N*_B_ and *N*_AB_ are the number of available states. Herein, *N*_states_ for [*V*_O_-Al_Ti_] except for the spin contribution is approximated to be twice that of *V*_O_ considering the two adjacent Ti where Al can be introduced. Let us now consider the case where the isolated defects are formed during the annealing process at a high temperature (1400 K in the present case), and they associate with each other at a certain lower temperature. The initial concentration of *V*_O_ $\left(C_{A}^{\mathrm{ini}}\right)$ at 1400 K is determined by the self-consistent defect calculation under a given chemical potential condition, where Δμ_Al_ was determined to make the $C_{B}^{\mathrm{ini}}$ equal to 1% of the Ti site density in SrTiO_3_. Equation 8 is solved under the conditions that $C_{A}^{\mathrm{ini}}=C_{A}+C_{AB}\mathrm{and}C_{B}^{\mathrm{ini}}=C_{B}+C_{AB}$ are fixed.

The calculated association ratio of the oxygen vacancy is shown in Figures 7a,d and S11. While *V*o^+2^ is mostly associated with Al_Ti_ at low temperatures under the oxygen-poor limit (condition **a**), *V*_O_^+1^ and *V*_O_^0^ remain unassociated due to the excess amount of *V*_O_ relative to Al_Ti_^–1^ (1 at%). The initial concentration of *V*_O_^0^ (5.3 × 10^21^ cm^–3^) is much higher than that of Al_Ti_^–1^ (1.7 × 10^20^ cm^–3^). To increase the association ratio at the oxygen-poor limit, the initial amount of Al_Ti_^–1^ could be increased. However, ∼80% of *V*_O_^0^ is still unassociated even when 5 at% of Al_Ti_^–1^ is introduced (Figure 7c). On the other hand, when the concentration of *V*_O_^0^ is lower under more oxygen-rich conditions (condition **h**), all the *V*_O_ species are associated with Al_Ti_^–1^ (1 at%) as shown in Figure 7d–f. The effectiveness of Al doping therefore depends on the atmosphere and the synthetic temperature.

**Figure 7:**
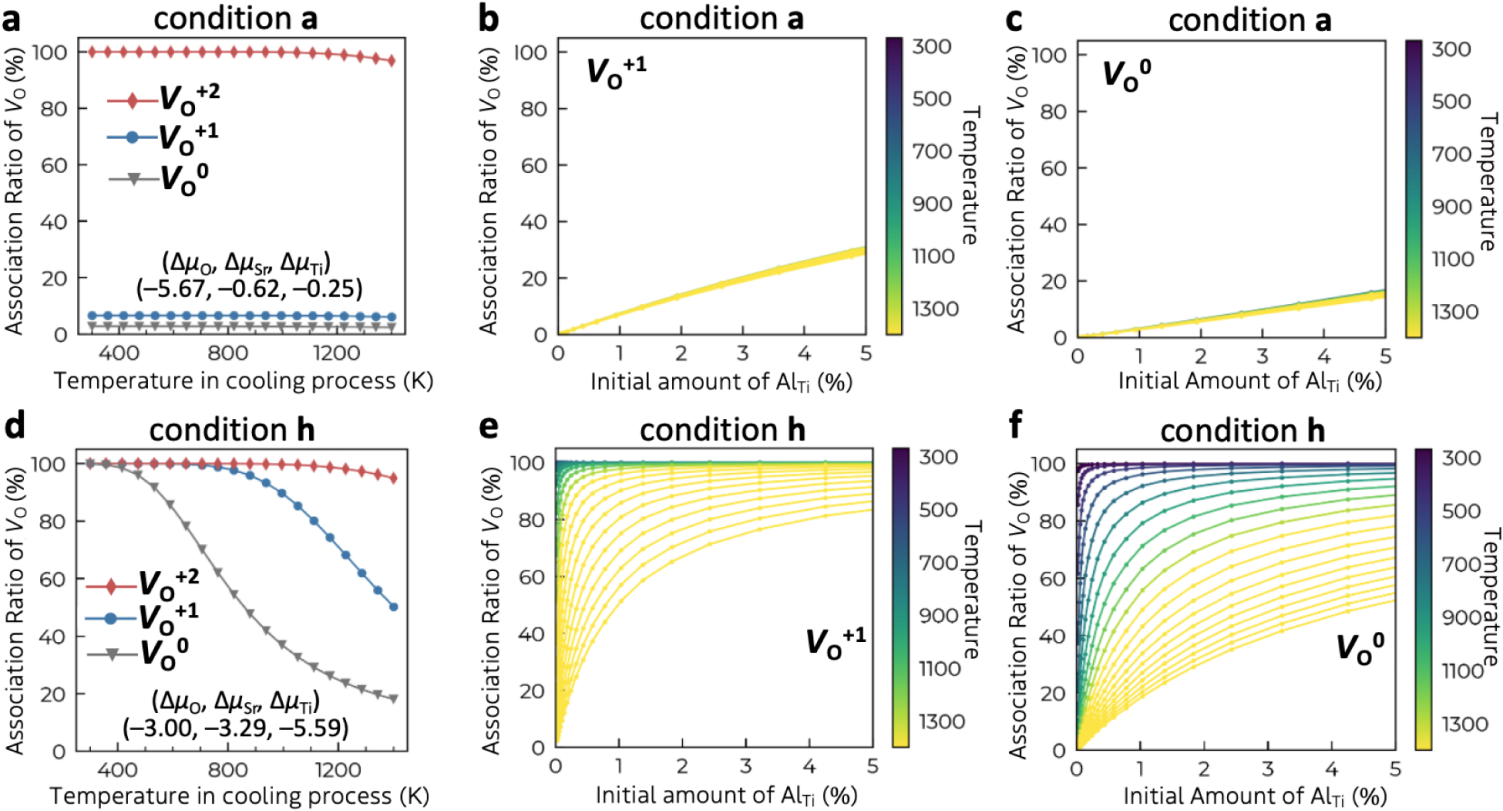
(a, d) Ratio of the concentration of the [*V*_O_-Al_Ti_]^0^ complex to the initial concentration of *V*_O_ (i.e., the association ratio) under condition **a** (oxygen poor limit) and condition **h** (Δμ_O_ = – 3 eV). We considered the case where the isolated defects are formed during the annealing process at 1400 K, and they associate with each other at a certain lower temperature. The initial concentration of Al_Ti_ at 1400 K is fixed to 1 at%. Dependency of *V*_O_ association ratio on the initial concentration of Al_Ti_ at (b, c) condition **a** and (e, f) condition **h**.

Although the precise chemical potential during actual synthesis is difficult to determine, the influence of oxygen partial pressure within the ideal gas approximation (Figure S8) shows that even a high vacuum condition (∼10^–12^ atm) provides Δμ_O_ of ∼−3.5 eV at 1400 K. In addition, the experimentally observed downward $\varepsilon_{F}$ shift with Al doping (0.5 eV) agrees with the downward shift at condition **h** (i.e.**,** Sr-rich condition with Δμ_O_ = **–**3 eV) of ∼0.4 eV, as shown in Figure 6e. Therefore, the actual synthesis condition might be more oxygen-rich than the oxygen-poor limit (Δμ_O_ of −5.7 eV), and nearly all the *V*_O_ can be associated with Al_Ti_ even when the Al doping amount is 1 at% (Figure 7d for condition **h**).

The electronic states of *V*_O_ change upon association with Al_Ti_. The in-gap states observed in *V*_O_^+1^ and *V*_O_^0^ are removed regardless of the charge state (Figures 8 and S12), making the trap level shallower (Figure 6a,d). As mentioned above, the in-gap state originates from the Ti 3d–Ti 3d interaction across the oxygen vacancy (Figure 4). Here, Al (3s^2^p^1^) does not possess d-orbitals as valence orbitals. Therefore, replacing one Ti adjacent to the oxygen vacancy with Al deactivates the Ti 3d–Ti 3d interaction, eliminating the in-gap state. The Al–Ti distance is larger than Ti–Ti distance in *V*_O_^+1^ and *V*_O_^0^, reflecting the absence of the bonding interaction between them. While the isolated *V*_O_ acts as a carrier capture center that can deteriorate the photocatalytic activity (Figure 5), the defect complex removes the in-gap states. This removal of trapping and recombination channels explains the significant photocatalytic activity enhancement in Al-doped SrTiO_3_ observed experimentally.^14,23^

**Figure 8:**
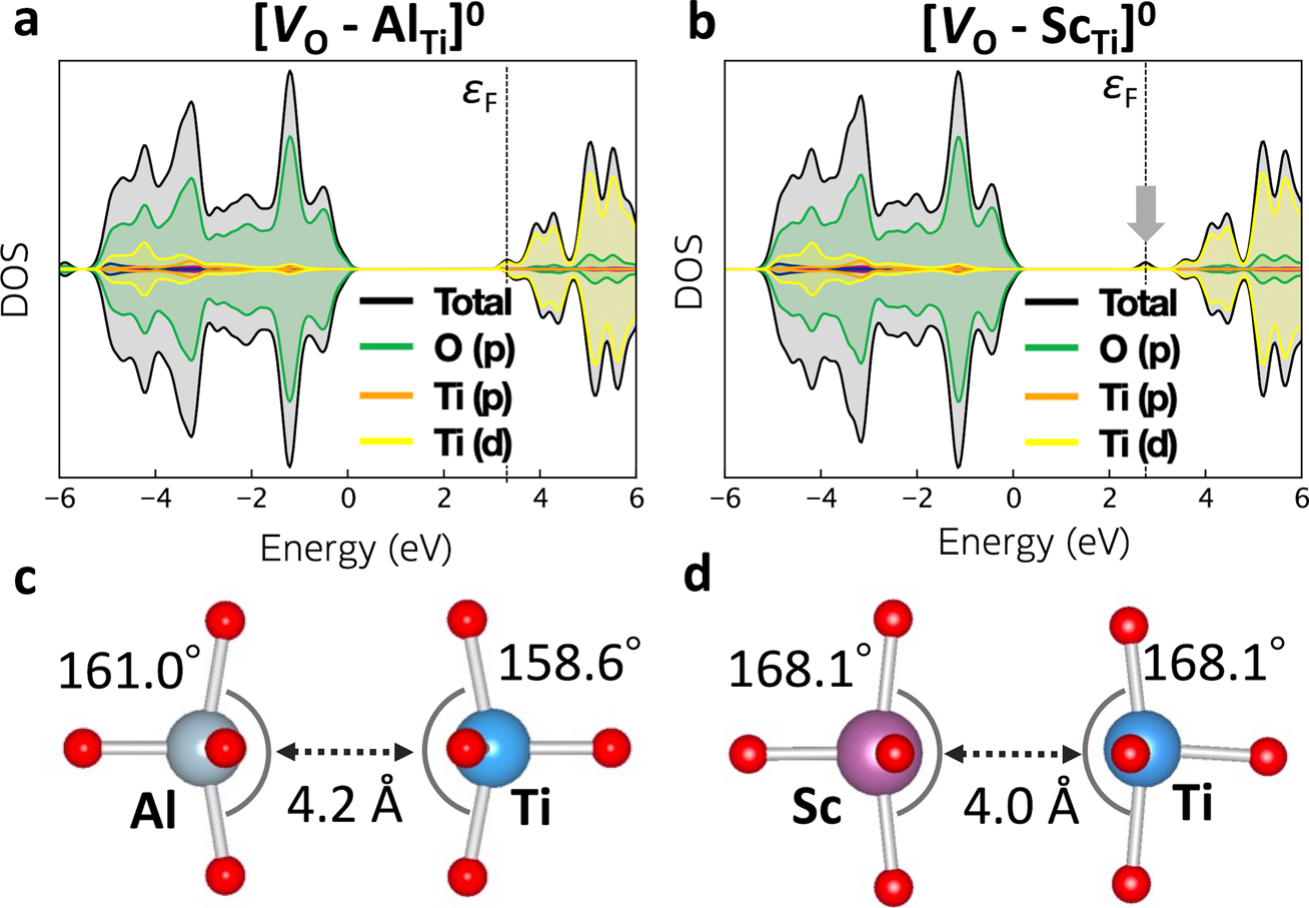
Electronic density of states for SrTiO_3_ containing defect complex (a) [*V*_O_-Al_Ti_]^0^, (b) [*V*_O_-Sc_Ti_]^0^ with (c, d) the respective atomic geometries around the oxygen vacancy.

Previous studies have stressed that the key to improving the photocatalytic activity is the lower valency of Al^3+^ compared to Ti^4+^. This conclusion is supported from the improved activity by Al^3+^, Ga^3+^, and Mg^2+^ and the lowered activity by Ta^5+^.^6^ However, these cations can be classified also by the absence (Ga (4s^2^p^1^, Mg (3s^2^)) or presence (Ta (5d^3^6s^2^)) of the d valence orbitals. We propose that the frontier orbital compatibility is also a key factor. To illustrate this point, Sc (3d^1^4s^2^) doping was also investigated. Sc^3+^ has a lower valency than Ti^4+^ but with d valence orbitals. The negative complex binding energy (Δ*E* = −0.36 eV) shows that Sc_Ti_^+1^ associates favorably with *V*_O_^–1^, as indicated previously.^82^ As shown in Figure 8b, Sc_Ti_ does not eliminate the localized in-gap state when complexed with *V*_O_. The Sc 3d can interact with Ti 3d orbitals as suggested by the shorter Sc–Ti distance compared to Al–Ti (Figure 8c, d) and the emergence of an associated in-gap electronic state. We note that the lower valence of the dopant is also required to provide an electrostatic driving force for complexation.

## Conclusion

The oxygen vacancy in SrTiO_3_ results in a detrimental defect state that traps photoexcited electrons. The origin of the trap state is a Ti 3d-Ti 3d interaction across the oxygen vacancy, whose bonding combination is stabilized within the band gap. We conclude that Al is preferentially incorporated substitutionally on Ti sites. Isolated Al_Ti_ acts as a hole capture site. However, the beneficial effect of Al emerges via the formation of [*V*_O_-Al_Ti_] complexes that eliminate the trap states. Replacing one Ti with Al without d valence orbitals deactivates the d-d interaction across the oxygen vacancy from which the in-gap state is derived. While the lower valency of Al helps to form the complex, it is insufficient to passivate the in-gap state. This is illustrated by Sc doping, where the Sc 3d–Ti 3d interaction does not eliminate the vacancy-induced in-gap states. The absence of d valence orbitals in the dopant is found to be key. The present study not only reveals the functional mechanism of Al doping to SrTiO_3_, but also provides a strategy from the viewpoint of orbital interactions to “externally” impart the defect-tolerance to even conventional semiconductors by passivating in-gap trap states that hinder solar-to-electrical energy conversion.

## Experimental Section

### First-Principles Calculations

All first-principles calculations were performed using the projector augmented-wave (PAW) method^83^ under three-dimensional periodic boundary conditions as implemented in the Vienna Ab Initio Simulation Package (VASP).^84–86^ PAW data sets with radial cutoffs of 1.3, 1.2, 0.8, and 1.0 Å for Sr, Ti, O, and Al, respectively, were employed, where Sr (4s, 4p, 5s), Ti (3p, 4s, 3d), O (2s, 2p), and Al (3s, 3p) were treated as valence electrons. The HSE06 hybrid exchange-correlation functional^36^ was used, which provides a good agreement in the band gap with experiment (Table S1). The plane-wave cutoff energy and Γ-centered *k*-point mesh were sequentially increased using the vaspup2.0 package^87^ until the total energies from static calculations were converged to within 1 meV/atom. The given values were 600 eV and 5 × 5 × 5 for the 5-atom primitive cell of SrTiO_3_. The atomic positions were optimized until the Hellman–Feynman forces on each atom were below 0.01 eV Å^–1^. The energy convergence criterion was set to 10^–6^ eV. The ionic dielectric response was calculated by density functional perturbation theory^88^ using the Perdew–Burke–Ernzerhof (PBE) formulation of the generalized gradient approximation (GGA) functional,^89^ while the electronic contribution was calculated using the approach developed by Furthmüller et al.^88^ with HSE06 functional. Electronic band structure diagrams were generated and analyzed using the sumo package.^90^ Crystal orbital Hamilton populations (COHPs) were calculated using the LOBSTER package.^91^

### Defect Modeling

In the present study, we employed the supercell formalism for computing defect energetics. The formation energy of a defect *X* in charge state *q*, $E_{X,q}^{f}\left(\varepsilon_{F},\mu \right)$ , is given as^41–43^

9 $$E_{X,q}^{f}\left(\varepsilon_{F},\mu \right)=E_{X,q}-\left[E_{H}+\underset{i}{\sum }n_{i}\mu_{i}+q\cdot \left(-\varepsilon_{F}\right)\right]+E_{\mathrm{corr}}\left(q\right)$$

where the total number of elements and electrons are conserved between *E_X,q_* and $\left[E_{H}+\underset{i}{\sum }n_{i}\mu_{i}+q\cdot \left(-\varepsilon_{F}\right)\right]$ . A 135-atom supercell from a 3 × 3 × 3 expansion of the cubic SrTiO_3_ conventional cell was used to minimize the interactions between the periodically repeated defects under three-dimensional periodic boundary conditions. Note that an odd supercell expansion is used for SrTiO_3_ to “lock” the cubic structure^63^ by restricting octahedral tilting^92^ (see Figures S13 and S14). Local relaxation of the rattled bulk supercell with fixed volume does not find a lower energy structure. While a better approximation of the cubic structure, and response to defect formation, could be obtained from structural ensembles through molecular dynamics simulations, the computational cost would be prohibitively expensive at the present time. The ShakeNBreak approach^48,49^ was used to initially generate Γ-point-only relaxations for each defect with 10 different local distortions, where more stable defect structures than the unperturbed ones were often found (Figures S15–S17). The ground state structure found in the initial relaxation was further optimized with a 2 × 2 × 2 *k*-point mesh to obtain the total energy of a defect structure. The extended Freysoldt–Neugebauer–Van de Walle (eFNV) correction scheme was also employed,^44,45^ using a calculated static dielectric constant of 6.33.

The formation energy of each defect species ${Ε}_{X,q}^{f}\left(\varepsilon_{F},\mu \right)$ depends on the Fermi level according to eq 9); ${Ε}_{X,q}^{f}\left(\varepsilon_{F},\mu \right)\propto q\varepsilon_{F}$ . At a certain Fermi level *ε*_*F*_and temperature T , the concentration of defect X in charge state q $\left(C_{X,q}\right)$ including the effect of the defects competing for the same site is given by^93,94^

$$C_{X,q}=N_{\mathrm{site}}^{X}\frac{N_{\mathrm{config}}^{X,q}\exp\left(-\frac{{Ε}_{X,q}^{f}\left(\varepsilon_{F},\mu \right)}{k_{B}T}\right)}{1+\underset{X^{\prime }}{\sum }\underset{q^{\prime }}{\sum }N_{\mathrm{config}}^{X^{\prime },q^{\prime }}\exp\left(-\frac{{Ε}_{X^{\prime },q^{\prime }}^{f}\left(\varepsilon_{F},\mu \right)}{k_{B}T}\right)}$$ 10

where $N_{\mathrm{site}}^{X}$ is the density of lattice sites on which the defect *X* can form, while *N*_config_ is the number of equivalent configurations (degeneracy) at each site.^95,96^ *N*_config_ is estimated by the point symmetry change around the defect site and the spin degeneracy (Table S4).^39^ The sum in the denominator is performed over all defects (with charge *q*') that compete for the same site as defect *X*. Note that when the concentration of defects is small enough (i.e., ${Ε}_{X^{\prime },q^{\prime }}^{f}$ ≫ *k*_B_T), eq 10 reaches a Boltzmann distribution:

11 $$C_{X,q}=N_{\mathrm{site}}^{X}N_{\mathrm{config}}^{X,q}\exp\left(-\frac{{Ε}_{X,q}^{f}\left(\varepsilon_{F},\mu \right)}{k_{B}T}\right)$$

Although the Fermi level $\varepsilon_{F}$ is a free variable in eq 9), it is fixed by the charge neutrality condition:

$$p-n+\underset{X}{\sum }\underset{q}{\sum }qC_{X,q}=0$$ 12

where p and n are the concentrations of free holes and electrons in the valence and conduction bands. Not only $C_{X,q}$ C_*X*,*q*_ but n and p also depend on the Fermi level:

$$n=\int_{\varepsilon_{C}}^{\infty }f_{e}\left(\varepsilon \right)\rho \left(\varepsilon \right)d\varepsilon ;,f_{e}\left(\varepsilon \right)=\frac{1}{1+\exp\left(\frac{\varepsilon -\varepsilon_{F}}{k_{B}T}\right)}$$ 13

$$p=\int_{-\infty }^{\varepsilon_{V}}f_{h}\left(\varepsilon \right)\rho \left(\varepsilon \right)d\varepsilon ;,f_{h}\left(\varepsilon \right)=1-\frac{1}{1+\exp\left(\frac{\varepsilon -\varepsilon_{F}}{k_{B}T}\right)}$$ 14

where $\varepsilon_{V}$ , $\varepsilon_{C}$ are the VBM and CBM energies, ρ(ε) is the electronic density-of-states, and $f_{e}\left(\varepsilon \right)$ is the Fermi–Dirac distribution function. As $C_{X,q}$ , p and n depend on $\varepsilon_{F},\varepsilon_{F}$ is self-consistently determined by eq 12.^50,51^

The carrier and defect concentrations as a function of anneal temperature and chemical potential were estimated using the frozen defect approximation to mimic a synthetic condition where the material is annealed under elevated temperatures and cooled rapidly to room temperature.^51,52^ Defects are formed at the elevated annealing temperature but remain during cooling due to the assumed large kinetic barriers for diffusion and annihilation. Using the total concentration of each defect fixed to that at the annealing temperature, $\varepsilon_{F}$ is self-consistently calculated at room temperature (*T* = 300 K) where relative populations of defect charge states and carrier concentrations are re-equilibrated. The temperature dependence of the band gap (at annealing temperatures) was approximated using the experimentally reported band gap renormalization,^53^ and assuming symmetric shifts in the valence and conduction band edges while keeping the defect levels fixed. The doped Python package was used to manage all the defect calculations and analysis.^39^

The chemical potential of oxygen μ_O_ (*T*, *P*_*r*_) is obtained based on the results of the HSE06 hybrid functional calculation of an O_2_ molecule (triplet state) in a supercell (30 Å × 30 Å × 30 Å), where *P*_r_ is the reference pressure (1 atm). The translational, electronic, rotational and vibrational contributions at each temperature are calculated by vaspkit.^97^ Then μ_O_(*T*, *P*) was obtained as follows:

15 $$\mu_{O_{2}}\left(T,P\right)=\mu_{O_{2}}\left(T,P_{r}\right)+k_{B}T\;\ln\frac{P}{P_{r}}$$

16 $$\mu_{O}\left(T,P\right)=\frac{1}{2}\mu_{O_{2}}\left(T,P\right)$$

### Nonradiative Carrier Capture

The nonradiative carrier capture was simulated within the framework of multiphonon emission (MPE) using an extension of the method of Alkauskas et al.^73^ to account for anharmonicity. The carrier capture coefficient can be expressed using Fermi’s golden rule within linear electron–phonon coupling and first-order perturbation approximation, and one-dimensional configuration coordinate approach^73^

$$\mathrm{C̃}=\frac{2\pi }{\hbar }VgW_{if}^{2}\underset{m}{\sum }\omega_{m}\underset{n}{\sum }{\left|\langle \chi_{im}\mathrm{|Q}-Q_{0}|\chi_{fn}\rangle \right|}^{2}\;\delta \left(\Delta Ε+m\hbar \Omega_{i}-n\hbar \Omega_{f}\right)$$ 17

where *V* is the volume of the supercell, *g* is the degeneracy factor of the final state. $\omega_{m}$ is the thermal occupation of the vibrational state *m* of the initial state. Δ*E* is the energy difference between the initial and final state and Ω_{*i*,*f*}_ are the phonon frequencies of initial and final states. Q is defined as the one-dimensional configuration coordinate between the initial and final structures:

18 $$Q^{2}=\underset{\alpha }{\sum }M_{\alpha }\Delta R_{\alpha }^{2}$$

where $M_{\alpha }$ and $\Delta R_{\alpha }$ are the mass and the displacement of an atom α between the two configurations. The electron–phonon coupling matrix is expressed as

$$W_{if}=\left\langle \psi_{i}|\frac{\partial ĥ}{\partial Q}|\psi_{f}\right\rangle$$ 19

where $\psi_{j}$ and ĥ are the single-particle wave function and generalized Kohn–Sham Hamiltonian with the HSE06 hybrid functional, which is computed using nonrad^76^ within PAW formalism. CarrierCapture.jl^74,75,98^ was used to fit the potential energy surfaces (PES) and solve the 1D vibrational Schrödinger equation. The Sommerfeld parameter *s*(*T*)^99^ is considered to account for the Coulombic interaction between the electron/hole and charged defect. When a defect supercell used to calculate *W_if_* is charged, another scaling factor *f* is considered to correct the charged supercell effect.^76^ The scaled carrier capture coefficient is given as

20 C=s(T)fC̃

The capture cross-section is given by

21 σ=C/⟨v⟩

where ⟨v⟩ = $\sqrt{3k_{B}T/m^{*}}$ is the average thermal velocity of the carrier and $m^{*}$ is the average effective mass of electron/hole.

## Supplementary Material



## Data Availability

All relevant data generated in the course of this work is freely available at 10.5281/zenodo.15493773.

## Notes

Additional theoretical calculation results, including the formation energy of every defect and transition level diagram at every chemical potential conditions (PDF)

## #

Department of Chemistry, Graduate School of Science, Kyoto University, Kitashirakawa Oiwake, Sakyo, Kyoto, Japan, 606-8502
