# Supplementary material for: Defect Tolerance via External Passivation in the Photocatalyst SrTiO3:Al
Source: J Am Chem Soc. 2025 Jun 23;147(26):23180–91. doi: 10.1021/jacs.5c07104 (PMC12232298; doi:10.1021/jacs.5c07104)
Supplement: Supplementary file 1 [file ja5c07104_si_001.pdf]

Supporting Information

Defect Tolerance via External Passivation in the Photocatalyst $SrTiO_3$:Al

Kanta Ogawa[1,2,3]*, Seán R Kavanagh[4], Fumiyasu Oba[2,3], and Aron Walsh[1]

[1]Department of Materials, Imperial College London, London SW7 2AZ, UK

[2]Materials and Structures Laboratory, Institute of Integrated Research, Institute of Science Tokyo, R3-7, 4259 Nagatsuta, Midori-ku, Yokohama 226-8501, Japan

[3]Laboratory for Materials and Structures, Institute of Innovative Research, Tokyo Institute of Technology, R3-7, 4259 Nagatsuta, Midori-ku, Yokohama 226-8501, Japan

[4]Harvard University Center for the Environment, Harvard University, Cambridge MA, USA

Table S1. The band gap calculated using different functionals

	Exp[a]	GGA	GGA+U (U = 7, J = 1)	PBE0	HSE06
direct	3.75	2.17	3.00	4.44	3.72
indirect	3.25	1.92	2.64	4.21	3.34

[a] *Journal of Applied Physics* **2001**, *90*, 6156

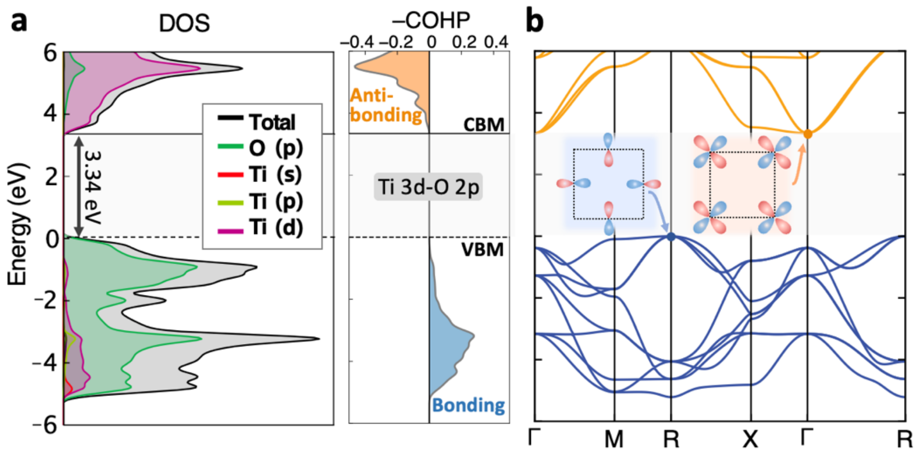

Figure S1. (a) Electronic density of states with crystal orbital Hamilton populations (COHP) for Ti 3d and O 2p. (b) Electronic band structure.

Table S2. Calculated formation energies of all competing phases.

Formula	Space Group	Energy above Hull (eV/atom)	Formation Energy (eV/fu)	Formation Energy (eV/atom)	k-points
$SrTiO_3$	*I*4/*mcm*	0.000	-17.881	-3.576	3x3x4
Sr	*P*6$_3$/*mmc*	0.000	0.000	0.000	8x8x4
Ti	*P*6/*mmm*	0.000	0.000	0.000	8x8x10
Al	$Fm\bar{3}m$	0.000	0.000	0.000	18x18x18
O_2	*C*2/*m*	0.000	0.000	0.000	3x3x3
SrO_2	*Pnma*	0.000	-6.756	-2.252	3x3x2
Ti_2O	$P\bar{3}m1$	0.000	-6.172	-2.057	8x8x4
Ti_2O_3	$R\bar{3}c$	0.000	-16.630	-3.326	3x3x3
TiO	$P\bar{6}2m$	0.000	-5.850	-2.925	5x5x7
TiO_2	$I4_1/amd$	0.000	-10.643	-3.548	3x3x3
$Sr_3Ti_2O_7$	*I*4/*mmm*	0.000	-42.049	-3.504	3x3x4
$SrAl_2O_4$	$P2_1$	0.000	-24.201	-3.457	2x2x2
$SrTi_3Al_8O_{19}$	*C*2	0.000	-108.400	-3.497	2x2x3
$SrTiO_3$	*I*4/*mcm*	0.000	-17.881	-3.576	3x3x4
$SrTiO_3$	$Pm\bar{3}m$	0.000	-17.880	-3.576	5x5x5
Ti	*P*6$_3$/*mmc*	0.002	0.002	0.002	8x8x5
$SrTi_{11}O_{20}$	$P\bar{1}$	0.002	-110.271	-3.446	2x2x1
TiO_2	*C*2/*m*	0.003	-10.634	-3.545	4x4x2
TiO_2	$I4_1/amd$	0.004	-10.631	-3.544	4x4x4
Sr	$Fm\bar{3}m$	0.005	0.005	0.005	12x12x12
Sr	$R\bar{3}m$	0.006	0.006	0.006	12x12x12
Sr	*P*6$_3$/*mmc*	0.007	0.007	0.007	9x9x3
$SrAl_2O_4$	$P6_3$	0.007	-24.151	-3.450	2x2x2
$SrAl_2O_4$	$P6_122$	0.012	-24.116	-3.445	3x3x1
Al	*P*6$_3$/*mmc*	0.013	0.013	0.013	13x13x4
$SrAl_2O_4$	*C*2	0.015	-24.096	-3.442	3x3x2
SrO_2	*I*4/*mmm*	0.020	-6.698	-2.233	4x4x4
Sr	$Im\bar{3}m$	0.022	0.022	0.022	10x10x10
O_2	*C*2/*m*	0.235	0.470	0.235	4x4x3
O_2	C2/*m*	0.244	0.487	0.244	4x4x3
O_2	$R\bar{3}m$	0.252	0.505	0.252	5x5x5

Table S3. Calculated formation energies (E^f) of all native point defects in $SrTiO_3$ under the O-poor condition (condition **a**).

Defect	q	$E_{X,q} - E_H$	$\sum_i n_i \Delta\mu_i$	E_{Corr}	$E^f_{X,q}$ (ε_F, = VBM)	$E^f_{X,q}$ (ε_F, = CBM)
V_{Sr}	0	11.70	-0.62	0.00	9.48	9.48
V_{Sr}	-1	14.80	-0.62	0.31	6.37	9.71
V_{Sr}	-2	18.01	-0.62	0.98	3.74	10.42
V_{Ti}	0	24.83	-0.25	0.00	15.07	15.07
V_{Ti}	-1	27.81	-0.25	0.32	11.85	15.19
V_{Ti}	-2	32.62	-0.25	1.04	10.88	17.6
V_{Ti}	-3	36.95	-0.25	2.08	9.73	19.75
V_{Ti}	-4	40.55	-0.25	3.50	8.24	21.60
V_O	2	-0.61	-5.67	0.66	0.83	-5.85
V_O	1	6.05	-5.67	0.15	0.46	-2.88
V_O	0	12.64	-5.67	0.00	0.39	0.39
Sr_{Ti}	0	16.66	0.37	0.00	9.12	9.12
Sr_{Ti}	-1	21.62	0.37	0.21	7.78	11.12
Sr_{Ti}	-2	25.71	0.37	0.81	5.94	12.62
Ti_{Sr}	2	-17.34	-0.37	0.83	4.05	-2.63
Ti_{Sr}	1	-11.87	-0.37	0.21	2.39	-0.95
Ti_{Sr}	0	-5.23	-0.37	0.00	2.31	2.31
Sr_i	2	-8.72	0.62	0.91	7.44	0.76
Sr_i	1	-2.08	0.62	0.32	6.97	3.63
Sr_i	0	4.62	0.62	0.00	6.84	6.84
Sr_i	4	-31.14	0.25	3.34	8.01	-5.35
Ti_{i1}	3	-25.40	0.25	2.09	6.00	-4.02
Ti_{i1}	2	-18.68	0.25	1.09	5.20	-1.48
Ti_{i1}	1	-11.88	0.25	0.33	4.72	1.38
Ti_{i1}	0	-5.17	0.25	0.00	4.59	4.59
Ti_{i1}	4	-31.19	0.25	3.38	8.01	-5.35
Ti_{i2}	3	-25.40	0.25	2.09	6.00	-4.02
Ti_{i2}	2	-18.68	0.25	1.09	5.20	-1.48
Ti_{i2}	1	-11.81	0.25	0.37	4.84	1.50
Ti_{i2}	0	-5.17	0.25	0.00	4.59	4.59
O_{i1}	0	-5.25	5.67	0.00	7.00	7.00
O_{i1}	-2	5.88	5.67	0.72	5.82	12.50
O_{i2}	0	-5.25	5.67	0.00	7.00	7.00
O_{i2}	-1	0.78	5.67	0.17	6.68	10.02
O_{i2}	-2	5.88	5.67	0.72	5.82	12.50

Table S4. N_{config} for each defect.

Defect	q	Site_Symm	Defect_Symm	g_Orient	g_Spin	N_{config}
V_{Sr}	0	O_h	C_{2v}	12	1	12
V_{Sr}	-1	O_h	C_1	48	2	96
V_{Sr}	-2	O_h	C_{3v}	8	1	8
V_{Ti}	0	O_h	C_s	24	1	24
V_{Ti}	-1	O_h	C_s	24	2	48
V_{Ti}	-2	O_h	C_s	24	1	24
V_{Ti}	-3	O_h	C_s	24	2	48
V_{Ti}	-4	O_h	C_{3v}	8	1	8
V_O	2	D_{4h}	C_{2v}	4	1	4
V_O	1	D_{4h}	C_s	8	2	16
V_O	0	D_{4h}	C_{2v}	4	1	4
Sr_{Ti}	0	O_h	C_1	48	1	48
Sr_{Ti}	-1	O_h	C_1	48	2	96
Sr_{Ti}	-2	O_h	C_3	16	1	16
Ti_{Sr}	2	O_h	C_s	24	1	24
Ti_{Sr}	1	O_h	C_s	24	2	48
Ti_{Sr}	Ti_{Sr}	O_h	C_s	24	1	24
Sr_i	2	C_s	C_{4v}	0.25	1	0.25
Sr_i	1	C_s	C_{4v}	0.25	2	0.5
Sr_i	0	C_1	D_{4h}	0.0625	1	0.0625
Sr_i	4	C_1	C_s	0.5	1	0.5
Ti_{i1}	3	C_s	C_{4v}	0.25	2	0.5
Ti_{i1}	2	C_1	C_{4v}	0.125	1	0.125
Ti_{i1}	1	C_{4v}	D_{4h}	0.5	2	1
Ti_{i1}	0	C_s	D_{4h}	0.125	1	0.125
Ti_{i1}	4	C_s	C_s	1	1	1
Ti_{i2}	3	C_1	C_{4v}	0.125	2	0.25
Ti_{i2}	2	C_1	C_{4v}	0.125	1	0.125
Ti_{i2}	1	C_1	Cs	0.5	2	1
Ti_{i2}	0	C_1	D_{4h}	0.0625	1	0.0625
O_{i1}	0	C_1	C_s	0.5	1	0.5
O_{i1}	-2	C_{2v}	D_{2h}	0.5	1	0.5
O_{i2}	0	C_1	C_s	0.5	1	0.5
O_{i2}	-1	C_1	D_{2h}	0.125	2	0.25
O_{i2}	-2	C_1	D_{2h}	0.125	1	0.125

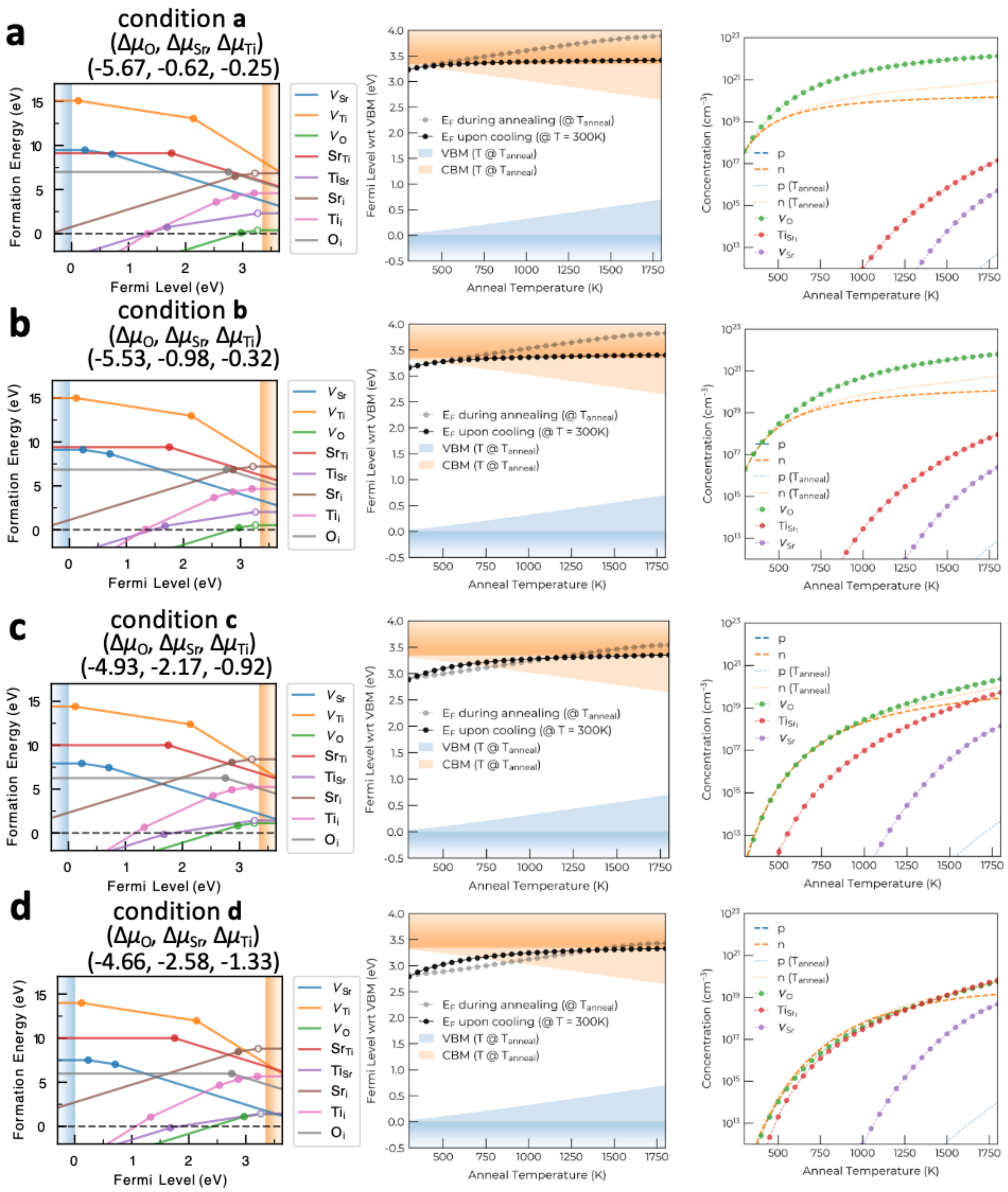

Figure S2. Transition level diagram of intrinsic defects in $SrTiO_3$ under O-poor condition at (a) condition **a**, (b) condition **b**, (c) condition **c**, (d) condition **d**), along with the calculated self-consistent Fermi level positions in undoped $SrTiO_3$ during annealing (gray) and upon cooling to room temperature (300 K; black), and room temperature carrier and defects concentration as a function of annealing temperature. Oxygen vacancy (V_O) is the highest concentration defect specie. Strontium-on-titanium anti-site (Ti_{Sr}) and strontium vacancies (V_{Sr}) are also relatively high in some concentrations. During the crystal growth of $SrTiO_3$:Al, Sr is rich in the $SrCl_2$ molten-salt solvent. The effect of the Sr chemical potential across conditions **a** – **d** is shown in Figure S4. Under Sr-rich condition, the formation of Ti_{Sr} and V_{Sr} is suppressed and V_O is the main defect species.

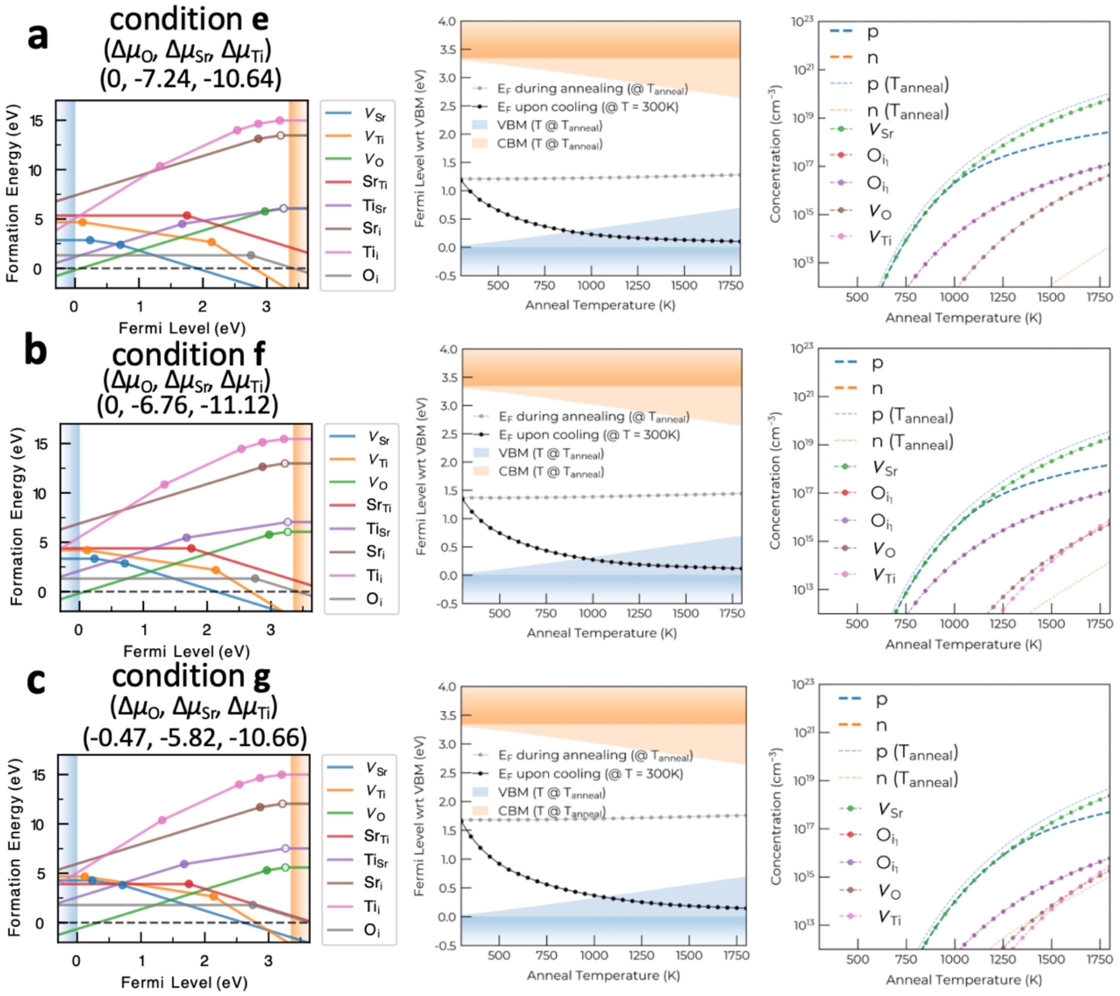

Figure S3. Transition level diagram of intrinsic defects in $SrTiO_3$ under oxygen-rich condition at (a) condition **e**, (b) condition **f**, (c) condition **g**, along with the calculated self-consistent Fermi level positions in undoped $SrTiO_3$ during annealing (gray) and upon cooling to room temperature (300 K; black), and room temperature carrier and defects concentration as a function of annealing temperature.

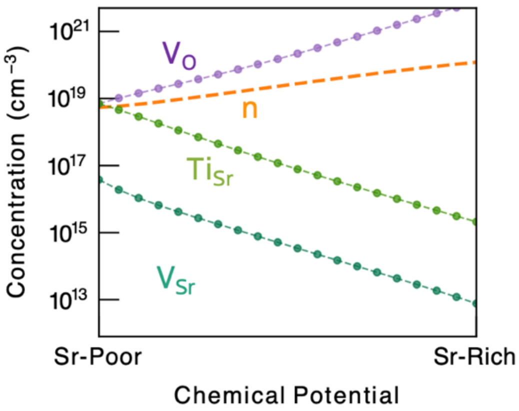

Figure S4. The effect of Sr chemical potential on the carrier and defects concentration under the oxygen-poor conditions (from conditions **d** to **a**).

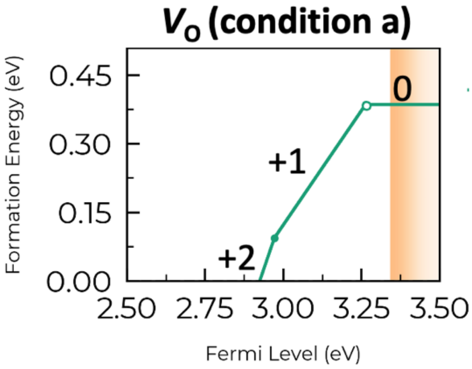

Figure S5. Transition level diagram of oxygen vacancy in $SrTiO_3$ at condition **a**.

Table S5. Charge transition level of each defects

Defect	Charges	eV from VBM
V_{Sr}	ε(0/-1)	0.236
V_{Sr}	ε(-1/-2)	0.71
V_{Ti}	ε(0/-1)	0.12
V_{Ti}	ε(-1/-4)	2.137
V_{Ti}	ε(+2/+1)	2.972
V_{Ti}	ε(+1/0)	3.263
Sr_{Ti}	ε(0/-2)	1.752
Ti_{Sr}	ε(+2/+1)	1.677
Ti_{Sr}	ε(+1/0)	3.264
Sr_i	ε(+2/+1)	2.868
Sr_i	ε(+1/0)	3.217
Ti_i	ε(+4/+3)	1.331
Ti_i	ε(+3/+2)	2.537
Ti_i	ε(+2/+1)	2.867
Ti_i	ε(+1/0)	3.203
O_i	ε(0/-2)	2.753

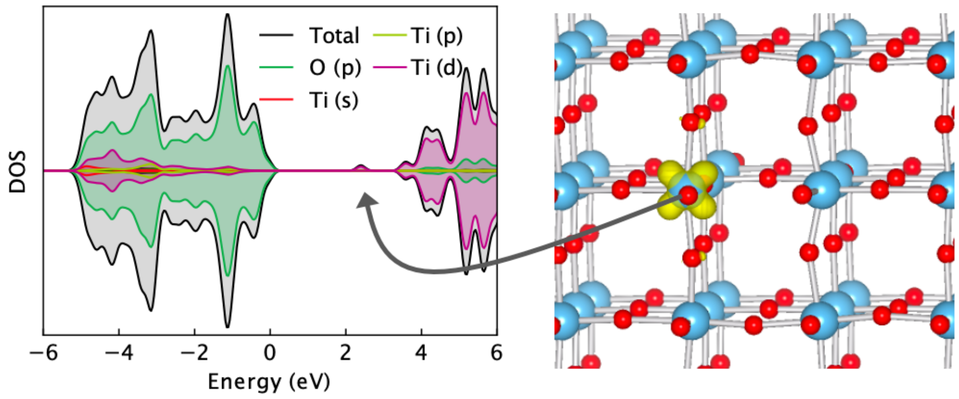

Figure S6. DOS and Electron density map of the polaronic state for V_O^{+1}. The structure with an electron localized on one Ti was provided by using Hubbard U approach, then the energy was calculated after removing the U value without the structural relaxation. The found structure is 0.26 eV/super cell higher in energy than the structure found by ShakenBreak approach. Note that without the structural fixation after removing the U, the structure was relaxed to the structure found by ShakenBreak approach shown in Figure 3d.

Table S6. Key parameters for the carrier capture coefficients in the transition of $V_O^{+2} \leftrightarrow V_O^{+1}$ and $Al_{Ti}^{0} \leftrightarrow Al_{Ti}^{-1}$.

Transition	ΔQ ($amu^{1/2}$Å)	Capture process	ΔE_b (meV)	g	W_{if} (eV/ $amu^{1/2}$Å)	s(300K)f	C (cm^3s^{-1})	σ (cm^2)
$V_O^{+2} \leftrightarrow V_O^{+1}$	1.82	electron	3.9	8	8.49×10^{-4}	72.23	5.6×10^{-8}	3.6×10^{-15}
		hole	2100	4	5.13×10^{-3}	0.0063	9.5×10^{-23}	7.4×10^{-30}
$Al_{Ti}^{0} \leftrightarrow Al_{Ti}^{-1}$	2.61	electron	543	1	8.60×10^{-4}	–	2.0×10^{-18}	1.3×10^{-25}
		hole	0.75	12	1.78×10^{-2}	19.1	1.1×10^{-5}	8.8×10^{-13}

Mass-weighted distortion ΔQ ($amu^{1/2}$Å), energy barrier ΔE_b (meV), degeneracy factor g of the final state, electron-phonon coupling matrix element W_{if}, and scaling factor $s(T)f$ at 300 K, along with calculated capture coefficient C (cm^3s^{-1}) and cross-section σ (cm^2) at 300 K. W_{if} was calculated via mean-square average of the W_{if} values with degenerated bands on CBM and VBM.

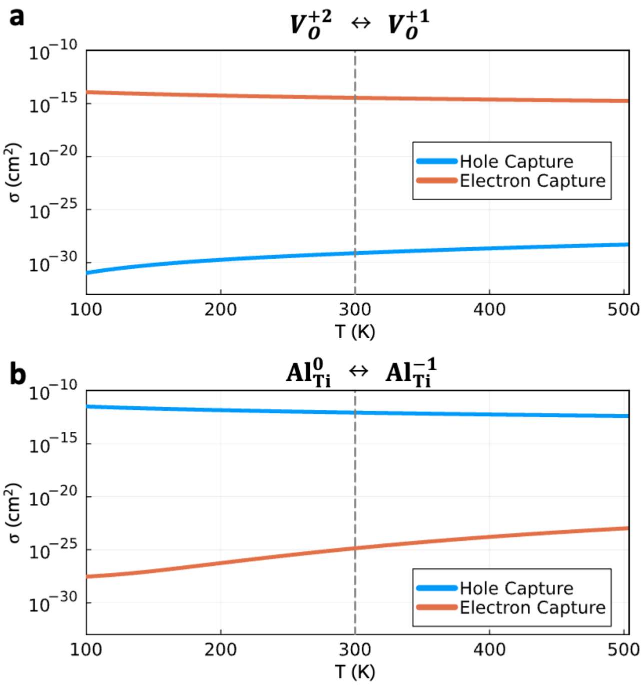

Figure S7. Carrier capture cross-sections for (a) V_O and (b) Al_{Ti} in $SrTiO_3$.

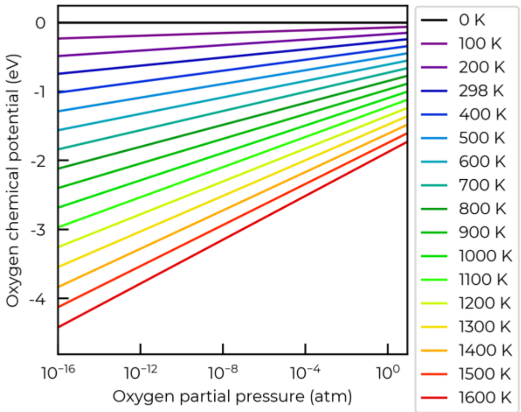

Figure S8. Oxygen chemical potential dependent on oxygen partial pressure within the ideal gas approximation. This suggest that the actual chemical potential condition during the flux-supported synthesis of $SrTiO_3$:Al (at ~1400 K) is from –2 to –4 eV, although the fugacity of O_2 gas in the molten $SrCl_2$ is not considered in Figure S8. Although the exact chemical potential condition during synthesis is difficult to determine, the defect property when $\Delta\mu_O$ = –3 eV (corresponding to 1400K and oxygen pressure of ~10^{-8} atm) and Sr is rich was calculated as shown in Figure 6, 7 and S9.

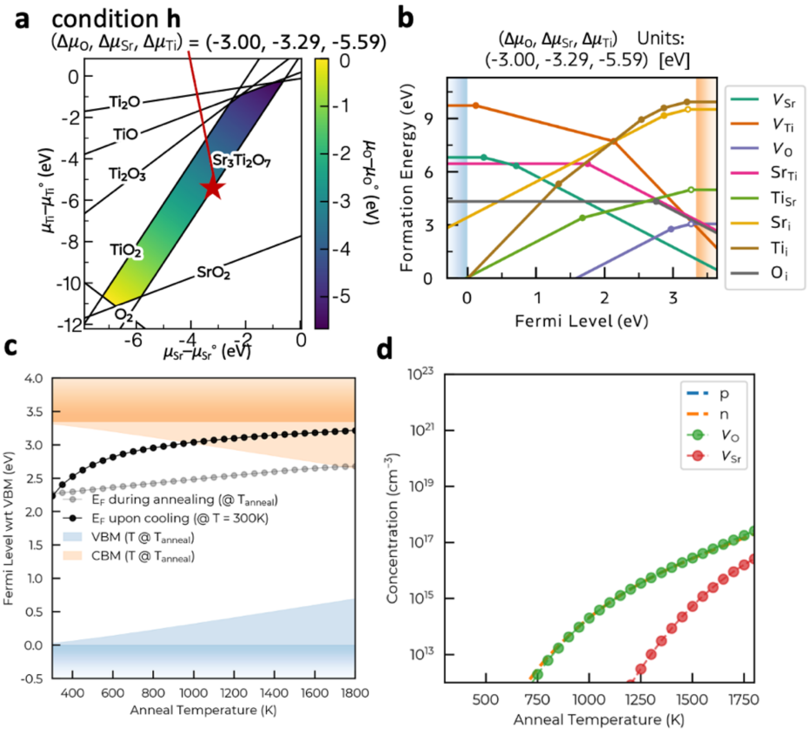

Figure S9. (a) Sr-rich condition with $\Delta\mu_O$ = –3 eV (red star; condition **h**) in the chemical potential stability region of $SrTiO_3$. (b) Transition level diagram of intrinsic defects in $SrTiO_3$ at this condition. (c) Calculated self-consistent Fermi level positions in undoped $SrTiO_3$ during annealing (gray) and upon cooling to room temperature (300 K; black). (d) Room temperature carrier and defect concentrations as a function of annealing temperature. These results show that the $SrTiO_3$ exhibits n-type nature and the main defect species is V_O even with $\Delta\mu_O$ of –3 eV.

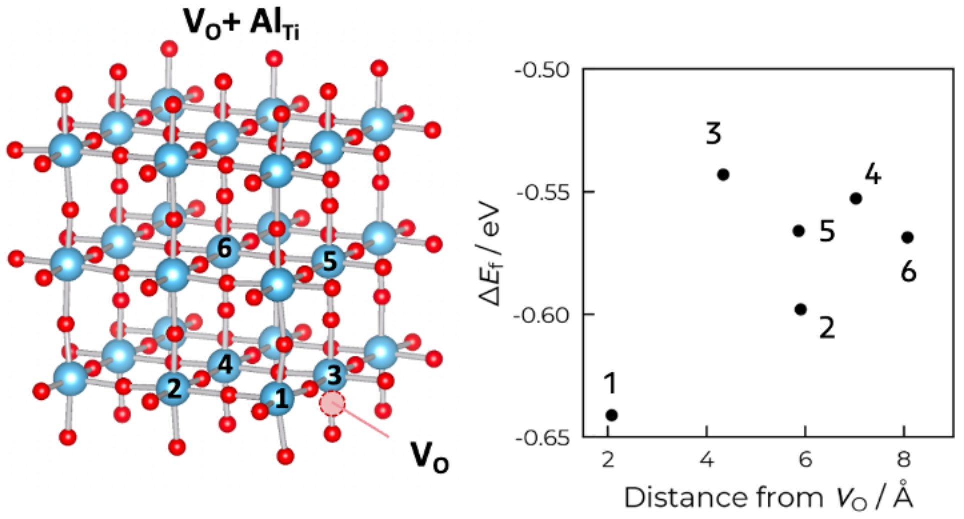

Figure S10. The site dependency of formation enthalpy of the [V_O^{+1}-Al_{Ti}^{-1}] complex from the isolated V_O^{+1} and Al_{Ti}^{-1}.

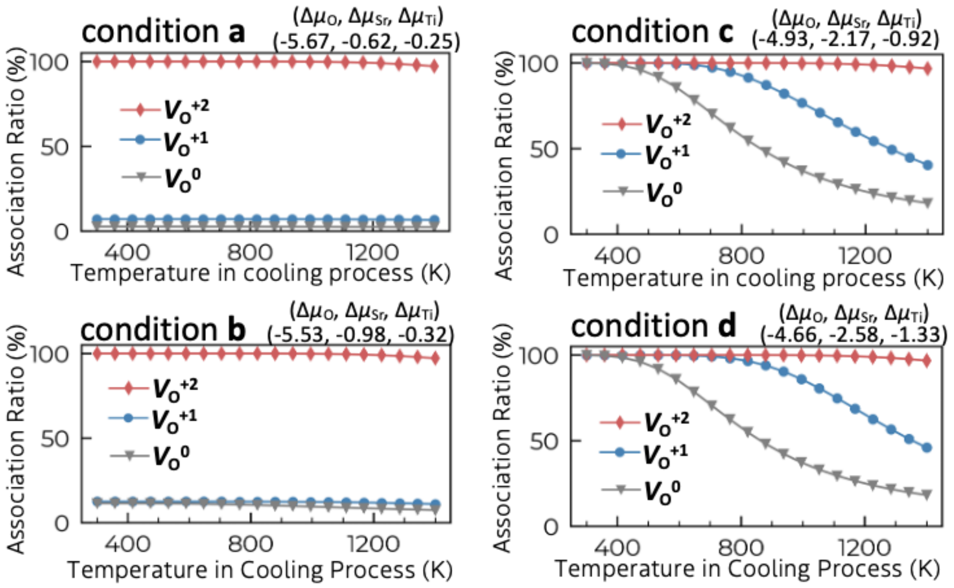

Figure S11. Ratio of the concentration of complexes [V_O-Al_{Ti}]^0 to the initial concentration of V_O (i.e., association ratio). The initial concentration of Al_{Ti} is fixed to 1 at%.

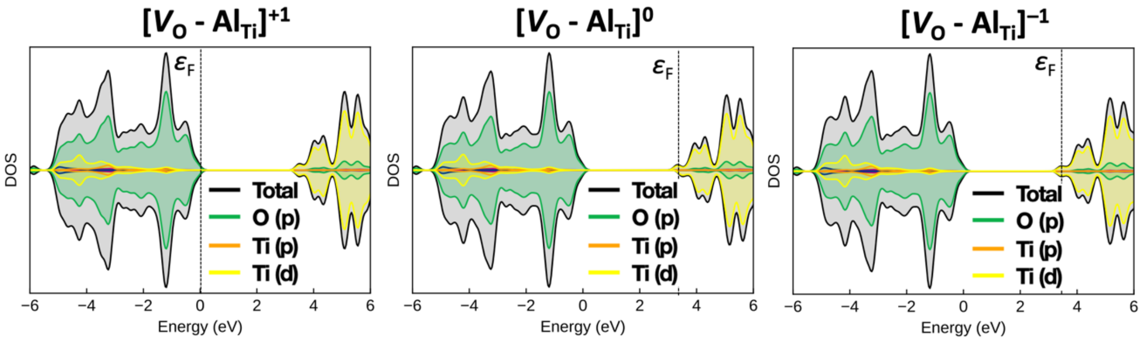

Figure S12. Electronic density of states for $SrTiO_3$ containing defect complex $[V_O\text{-}Al_{Ti}]^{+1/0/-1}$.

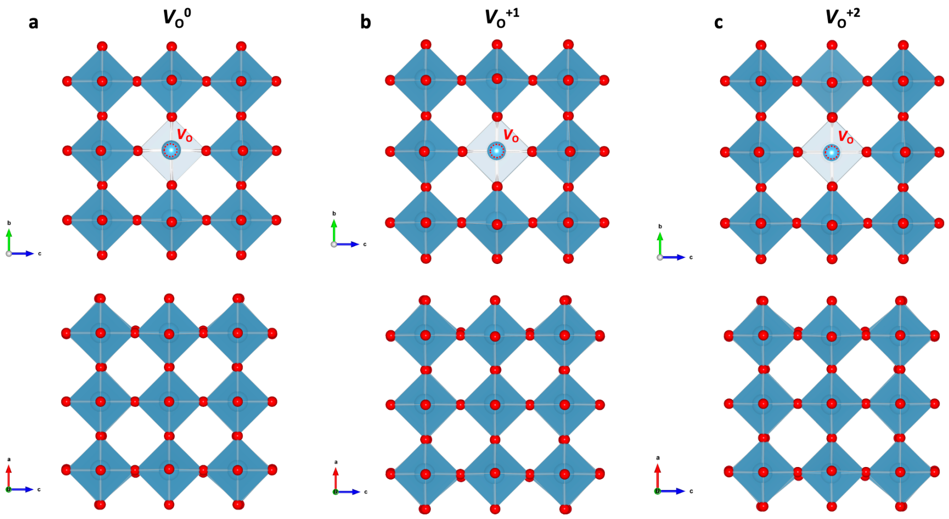

Figure S13. Crystal structures of a 3×3×3 supercell including (a) V_O^0, (b) V_O^{+1}, and (c) V_O^{+2}.

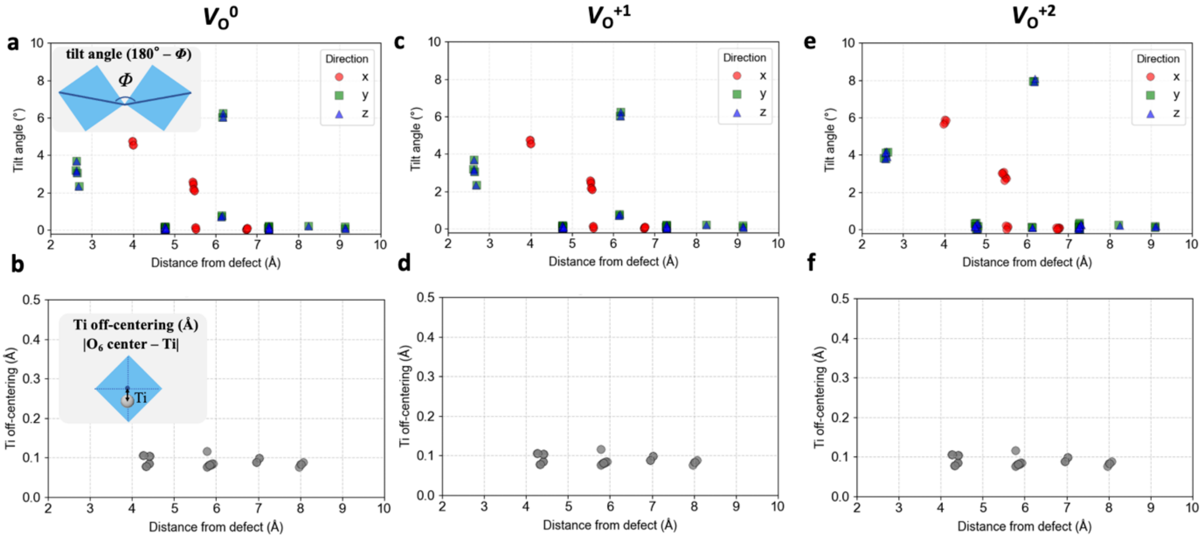

Figure S14. Octahedral tilt and Ti off-centering in the 3×3×3 supercell including (a, b) V_O^{+0}, (c, d) V_O^{+1}, (e, f) V_O^{+2} as a function of distance from the oxygen vacancy. The octahedral tilt angle is defined as 180° – *Φ*, where *Φ* is the angle between diagonally opposite O atoms in adjacent TiO_6 octahedra sharing a corner oxygen atom. Ti off-centering is calculated as the distance between the Ti atom and the mass center of its six surrounding oxygen atoms, where the Ti off-centering directly adjacent to the vacancy is excluded. The octahedral tilt angle remains small especially at sites far from the defect, as compared to those in anti-ferrodistortive-like octahedron rotation in cubic $SrTiO_3$ with an oxygen vacancy (~13°, *Adv*. *Mater*. 2013, 25, 86–90). It is worth noting that the relatively large tilt angle observed at ~6 Å from the vacancy arises from the two octahedra located directly along the line between vacancy periodic images. Herein, the tilt primarily originates from the displacement of an oxygen atom (~0.15 Å) that belongs to the octahedral unit including the vacancy, while the central oxygen at the tilt axis is slightly displaced (~0.06 Å), indicating that the overall octahedral tilting is limited within the current supercell. The Ti off-centering is also found to be small throughout the supercell.

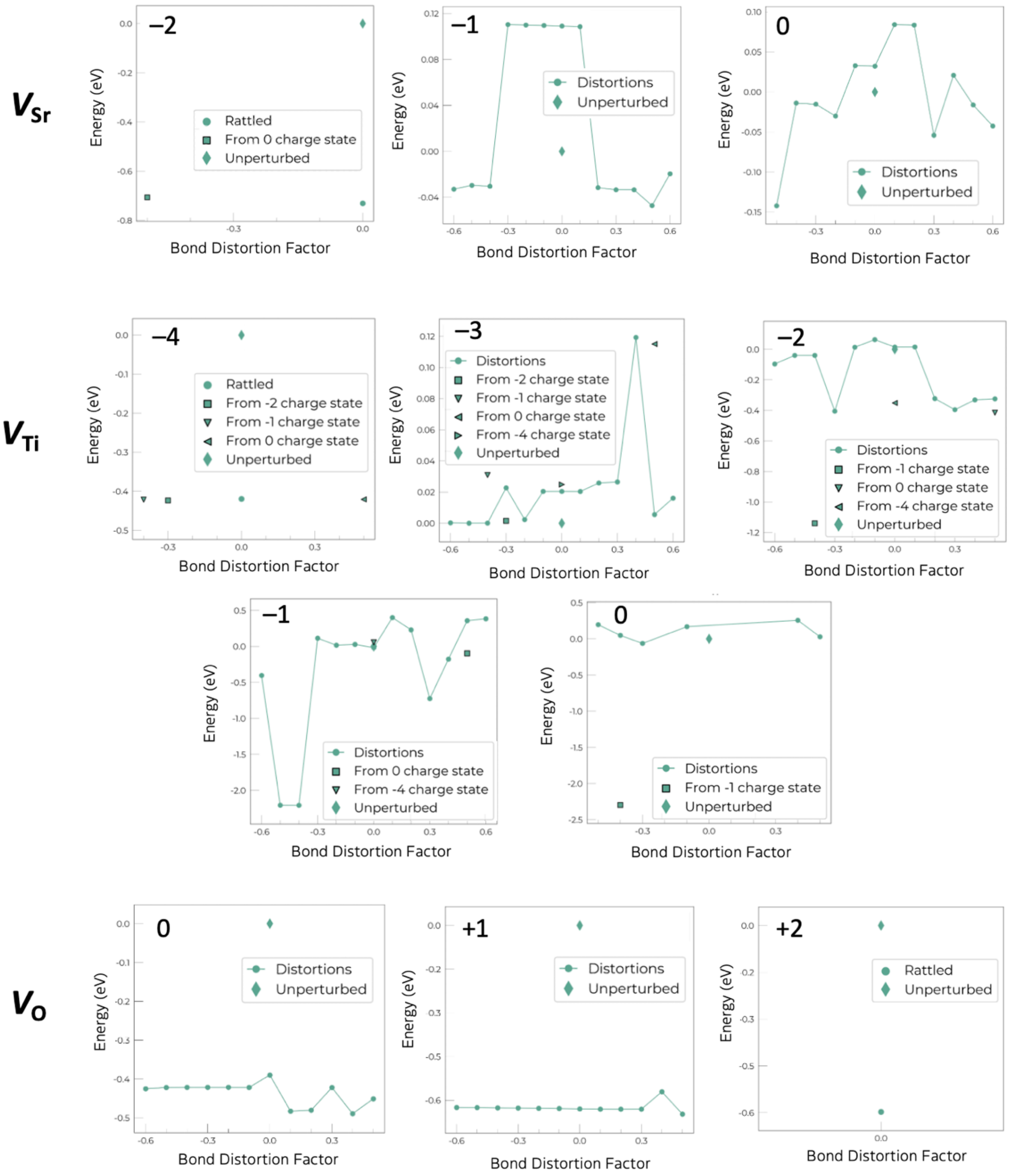

Figure S15. The relative energies of relaxed ShakeNBreak structures against the bond distortion factor for vacancies. The most stable structure of V_O^0 presents two electrons delocalized on the conduction band, which can be described as V_O^{+2} + 2 e_{CBM}. Therefore, the second stable structure was used for this case.

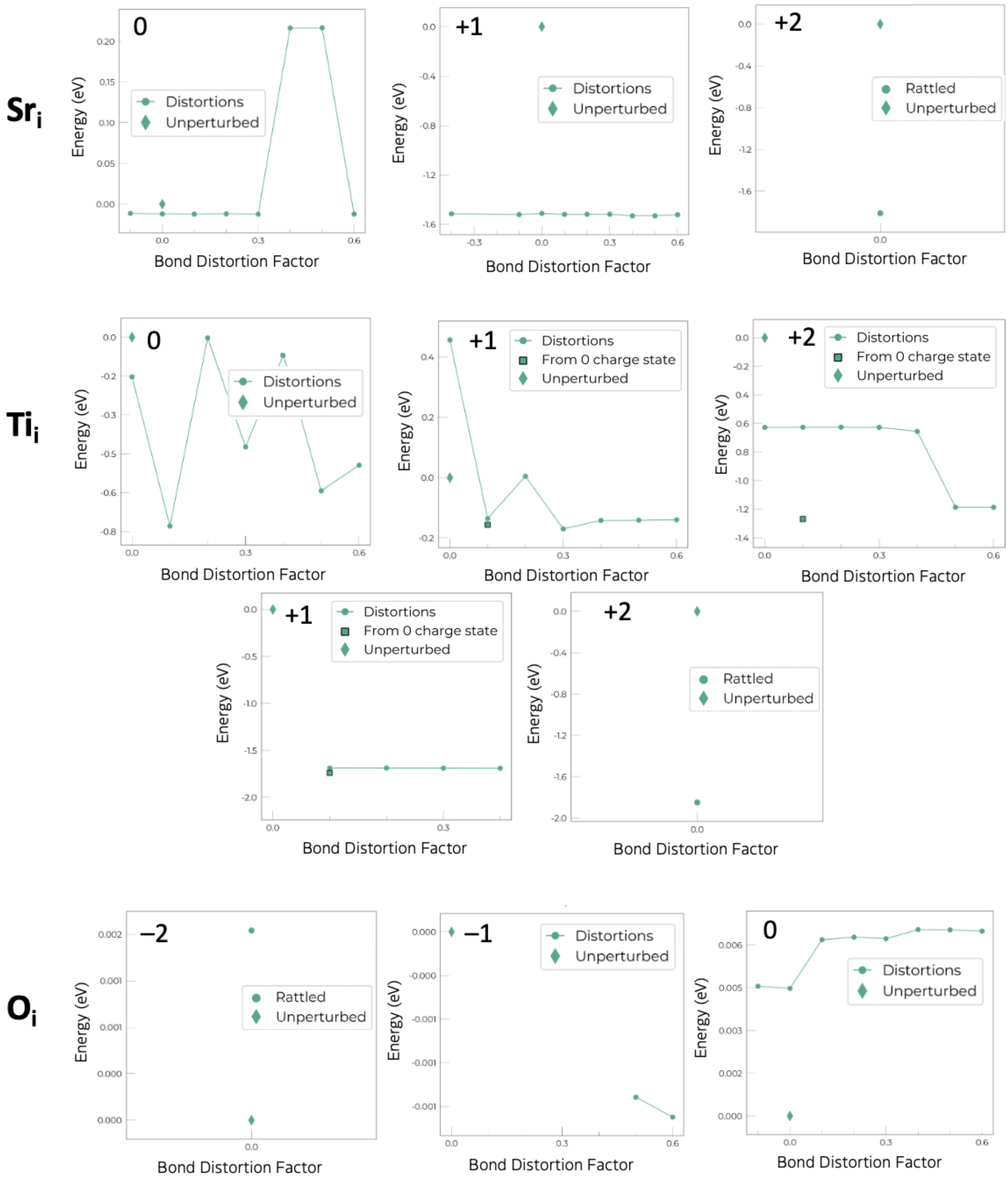

Figure S16. The relative energies of relaxed ShakeNBreak structures against the bond distortion factor for anti-sites.

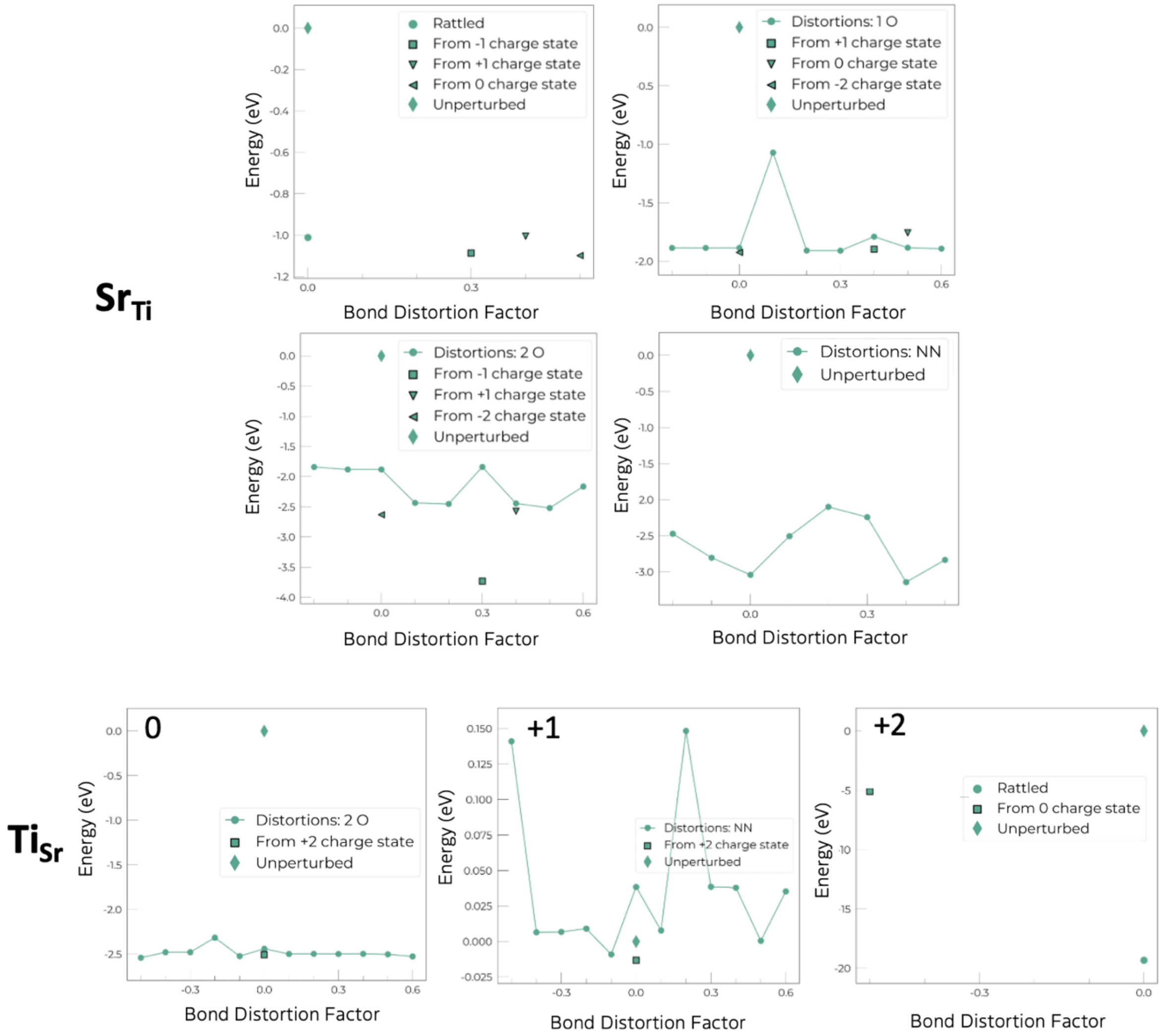

Figure S17. The relative energies of relaxed ShakeNBreak structures against the bond distortion factor for interstitials.
